# Mitochondrial DNA editing in potato through mitoTALEN and mitoTALECD: molecular characterization and stability of editing events

**DOI:** 10.1186/s13007-023-01124-9

**Published:** 2024-01-05

**Authors:** Alessandro Nicolia, Nunzia Scotti, Nunzio D’Agostino, Giovanna Festa, Lorenza Sannino, Gaetano Aufiero, Shin-ichi Arimura, Teodoro Cardi

**Affiliations:** 1 CREA, Research Centre for Vegetable and Ornamental Crops, via Cavalleggeri 25, 84098 Pontecagnano, SA Italy; 2 CNR-IBBR, Institute of Biosciences and BioResources, 80055 Portici, NA Italy; 3 Department of Agricultural Sciences, University of Naples Federico II, 80055 Portici, Italy; 4 Laboratory of Plant Molecular Genetics, Graduate School of Agricultural and Life Sciences, The University of Tokyo, Tokyo, Japan

**Keywords:** Mitochondrial DNA editing, MitoTALEN, MitoTALECD, Genome editing, Base editing, Chondriome, Double strand break, Homologous recombination, *Solanum tuberosum*

## Abstract

**Background:**

The aim of this study was to evaluate and characterize the mutations induced by two TALE-based approaches, double-strand break (DSB) induction by the *Fok*I nuclease (mitoTALEN) and targeted base editing by the DddA cytidine deaminase (mitoTALECD), to edit, for the first time, the mitochondrial genome of potato, a vegetatively propagated crop. The two methods were used to knock out the same mitochondrial target sequence (*orf125*).

**Results:**

Targeted chondriome deletions of different sizes (236–1066 bp) were induced by mitoTALEN due to DSB repair through ectopic homologous recombination of short direct repeats (11–12 bp) present in the target region. Furthermore, in one case, the induced DSB and subsequent repair resulted in the amplification of an already present substoichiometric molecule showing a 4288 bp deletion spanning the target sequence. With the mitoTALECD approach, both nonsense and missense mutations could be induced by base substitution. The deletions and single nucleotide mutations were either homoplasmic or heteroplasmic. The former were stably inherited in vegetative offspring.

**Conclusions:**

Both editing approaches allowed us to obtain plants with precisely modified mitochondrial genomes at high frequency. The use of the same plant genotype and mtDNA region allowed us to compare the two methods for efficiency, accuracy, type of modifications induced and stability after vegetative propagation.

**Supplementary Information:**

The online version contains supplementary material available at 10.1186/s13007-023-01124-9.

## Background

The genomes of plant organelles (plastomes and chondriomes for plastids and mitochondria, respectively) control, directly or interacting with nuclear genes, the morphology, physiology and agronomic performance of natural populations and crops [1, 2]. Among the best characterized traits affected by these effects, mutations in mitochondrial DNA (mtDNA) and/or incompatibility between mitochondrial and nuclear genes can lead to cytoplasmic male sterility (CMS), an important trait in hybrid seed production.

Plastids and mitochondria are usually inherited in a uniparental (maternal) way in higher plants [2], avoiding sexual recombination of the respective genomes. In addition, the high copy number of plastomes and chondriomes, the presence of gene conversion and several other molecular mechanisms contribute to preserving genome integrity and maintaining a low mutation rate [1, 3]. Therefore, limited variability is generally available for genetic studies as well as for breeding. Cytoplasmic genetic variability can affect several field performance parameters [4–7], with a low level of diversity that could potentially lead to disastrous consequences, as in T-CMS maize hybrids [8].

Unlike plastids, plant chondriomes exhibit a multipartite and variable structure derived from frequent recombinations and rearrangements based on direct and inverted repeats of different sizes [9, 10]. This phenomenon has been exploited in somatic (cy)hybridization, which has allowed the generation of novel variation useful to produce new phenotypes for genetic improvement as well as pinpoint responsible mitochondrial open reading frames (orfs)/genes [9, 11–14]. Such approaches, however, are time-consuming and labor intensive and require the analysis of many recombinant mutants. On the other hand, contrary to plastids, no reliable methods for stable transformation are available for mitochondria in higher plants, limiting the possibility of a straightforward analysis of their genomes [3, 15, 16]. This makes the ability to directly modify mitochondrial sequences highly desirable.

As far as the editing of higher plant mitochondria is concerned, the use of CRISPR/Cas, although reported in nonplant systems with some controversy [17–19], is limited by the difficulties of importing guide RNAs in cytoplasmic organelles. The aim of this study is to evaluate two TALE-based approaches, the *Fok*I nuclease (mitoTALEN) and targeted base editing by the DddA (Double-stranded DNA deaminase toxin A) cytidine deaminase (mitoTALECD), to edit, for the first time, the mitochondrial genome of potato, a vegetatively propagated crop. Both methods have been developed in mammalian systems [18, 20, 21] and then successfully used for editing chondriomes or plastomes of some plant species, all sexually propagated [15, 22–29].

Here, we successfully used the mitoTALEN and mitoTALECD approaches to edit a mitochondrial target sequence (*orf125*), present in cultivated potato but not in other Solanaceous species, that is putatively involved in male sterility induction [10, 30]. The use of both methods in the same plant genotype and mtDNA region allowed us to compare their efficiency, accuracy, type of induced modifications and stability after vegetative propagation.

## Results

**Fig. 1:**
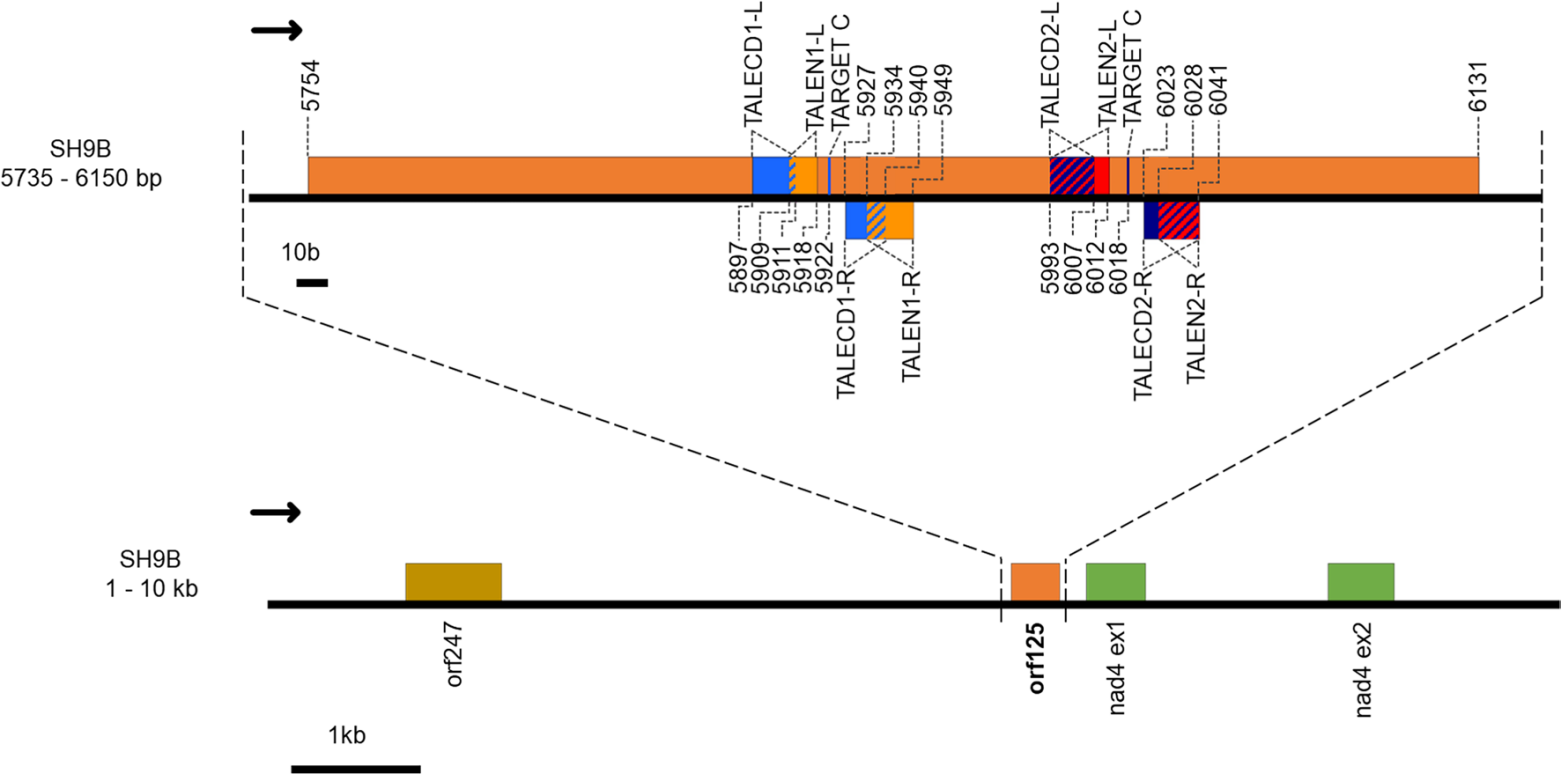
Genomic context of the target gene *orf125* in potato somatic hybrid SH9B. The genomic region reported corresponds to that between nucleotides 69,973 and 79,972 of Molecule 1 (GenBank MN104801.1) of the potato chondriome [10]. The positions of the TALEN1, TALEN2, TALECD1 and TALECD2 sequences, and those of the target Cs in base editing experiments, are also shown

The mitochondrial region of the potato somatic hybrid SH9B [31] investigated with TALEN and TALECD approaches is virtually identical (a single mismatch was found at position 76,769) to that between nucleotides 69,973 and 79,972 of Molecule 1 (GenBank MN104801.1) of the potato chondriome [10] (Fig. 1). The 378 bp long *orf125* sequence is between *orf247* and the first two exons of the *nad4* (NADH dehydrogenase subunit 4) gene. The two TALEN pairs (TALEN1-L/R and TALEN2-L/R) targeted *orf125* at nucleotide positions 156–196 and 240–288 starting from ATG, while the two TALECD pairs (TALECD1-L/R and TALECD2-L/R) targeted *orf125* at 144–187 and 240–288, respectively. Each pair of TALENs and TALECDs with mitochondrial targeting signals (the N-terminal presequence of the Arabidopsis mitochondrial ATPase delta-prime subunit) and the CaMV35S promoter are constructed to be expressed constitutively by T-DNA stably integrated into the nuclei and transported to function in the mitochondria (Additional file 1).

### MitoTALEN

#### Plant regeneration and genotyping

**Fig. 2:**
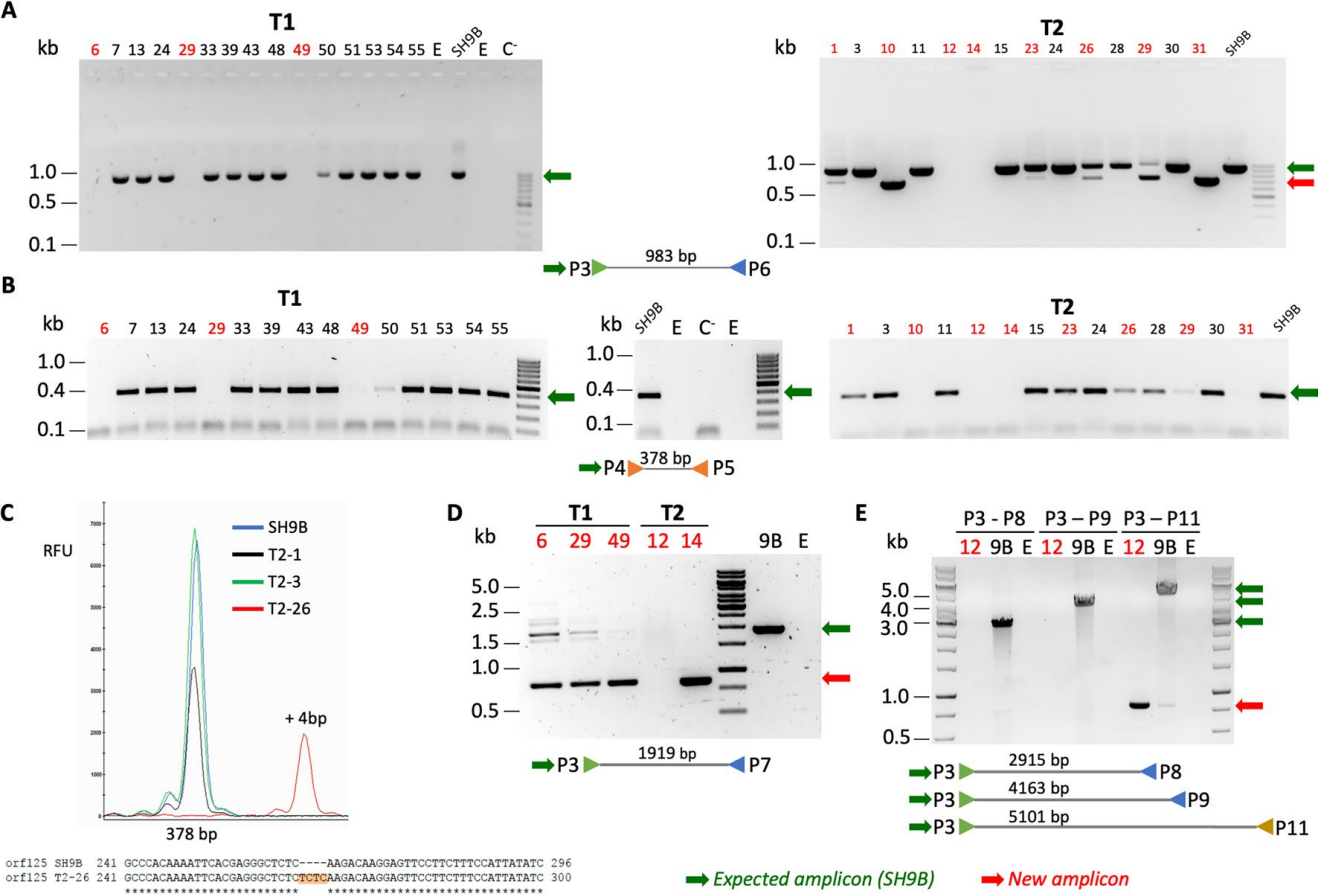
Amplifications of total DNA with various primer pairs in transgenic T1 and T2 plants and control SH9B. **A** P3–P6 primer pair; **B** P4–5 primer pair; **C** HRFA analysis in a sample of edited plants after amplification with P4–P5 primers and sequence of the T2–26 fragment showing the TCTC 4 bp insertion (highlighted); **D** P3–P7 primer pair; **E** P3–P8, P3–P9, P3–P11 primer pairs. 12, T2–12; 9B, SH9B. C^−^, negative control without DNA; **E** empty lane. The green and red horizontal arrows indicate the expected and variant amplicons, respectively

**Fig. 3:**
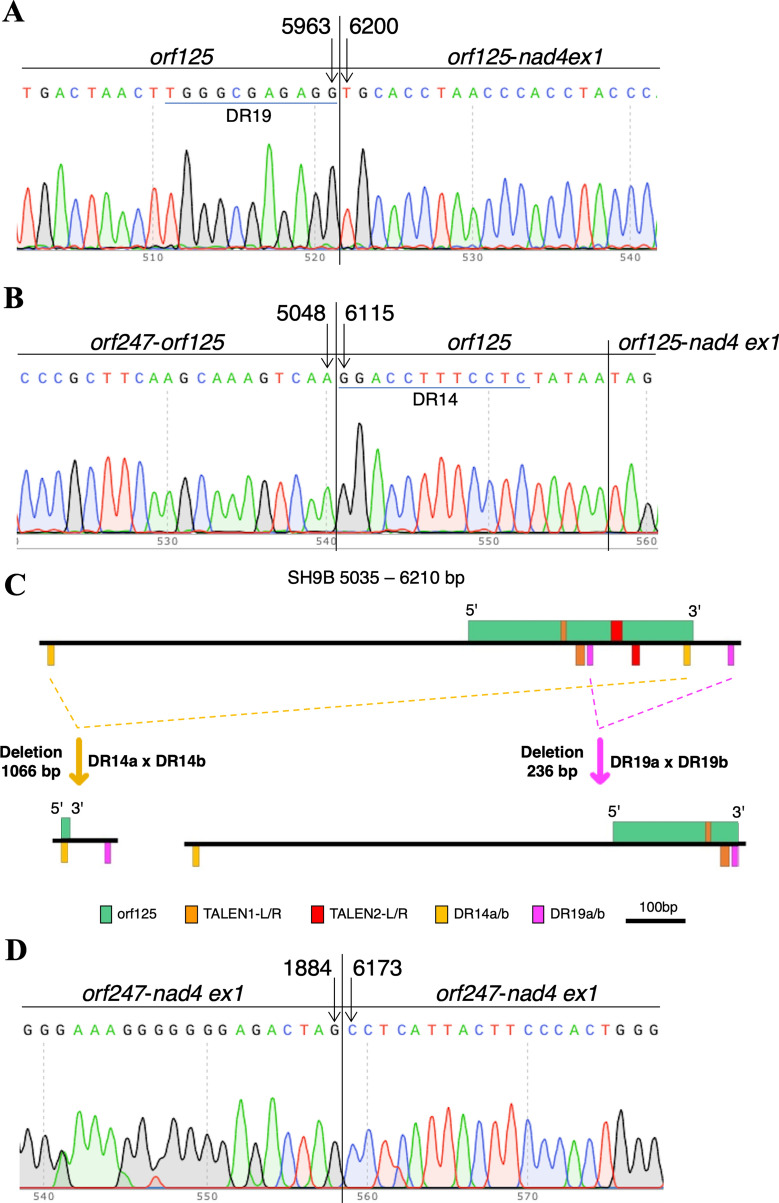
Induced deletions in a sample of representative edited plants (**A** T2–10, **B** T1–49, **D** T2–12). **A** Sequence of PCR products (P3–P6 primer pair) in plants showing a deletion of a 236 bp fragment between nucleotides 5966 and 6200. The DR19 sequence involved in recombination is underlined (see Additional file 5); **B** sequence of PCR products (P3–P7 primer pair) in plants showing a deletion of a 1066 bp fragment between nucleotides 5048 and 6115. The DR14 sequence involved in recombination is underlined (see Additional file 5); **C** graphical representation of the recombinations involving DR19 and DR14 sequences in plants shown in **A** and **B**, respectively; **D** sequence of PCR products (P3–P11 primer pair) in plants showing a deletion of a 4288 bp fragment between nucleotides 1884 and 6173

Twenty-one and 30 kanamycin-resistant shoots were obtained from 105 to 108 explants, respectively, in the two TALEN experiments. After PCR confirmation, *nptII*^*+*^ plants were tested with primers P3/P6 and P4/P5 (Fig. 2A, B and Additional file 2). In experiment T1 (TALEN1, Fig. 2A), all but three plants exhibited the expected amplicon size with both primer pairs. In contrast, T1–6, T1–29 and T1–49 plants showed no amplification. The same result was obtained in T2–12 and T2–14 plants in experiment T2 (TALEN2, Fig. 2A, B). In addition, after amplification with primers P3 and P6, six more plants showed a new fragment approximately two hundred bp shorter than expected, either singly (T2–10 and T2–31 plants) or in combination with wild-type (T2–1, T2–23, T2–26, T2–29), suggesting heteroplasmy for a putative deletion (Fig. 2A). Indeed, after sequencing, the new amplicon was found to contain a 236 bp deletion (Fig. 3A and Additional file 3A).

All plants showing an amplicon with primers P4 and P5 were subjected to high-resolution fragment analysis (HRFA), confirming the expected size of 378 bp in all but one case. Indeed, T2–26 also showed a 4 bp insertion (Fig. 2C) into the amplicon with a similar size to wild-type. The sequence confirmed a TCTC insertion (Fig. 2C), indicating the presence of multiple independent mutations in the mitochondrial genome.

To understand the cause of loss of P3/P6 and P4/P5 amplicons in T1–6, T1–29, T1–49, T2–12 and T2–14 plants, further PCR analyses were performed with primers that amplify the flanking genes or land on the intergenic region in combination with P3. The presence of *orf247* and the first two exons of *nad4* was confirmed in all plants (Additional file 4). Furthermore, a fragment approximately 1000 bp shorter than expected was obtained after amplification with P3/P7 primer pairs in all edited plants except T2–12 (Fig. 2D). T1–6, T1–29 and T1–49 also showed a wild-type band of varying intensity, in some cases barely visible, suggesting a heteroplamic status. After sequencing, the new P3/P7 amplicon was found to contain a 1066 bp deletion (Additional file 3B). T2–12 was subjected to further amplifications with P3/P8, P3/P9 and P3/P11, showing an amplicon of approximately 800 bp only in the latter case (Fig. 2E), suggesting a deletion of approximately 4300 bp. A very weak PCR product of the same size was visible in SH9B together with the expected product of approximately 5.1 kb, which, however, was significantly stronger.

#### Localization and recombination of direct repeats

With the aim of highlighting the origin of the deletions found in the edited plants, 19 direct repeats (10–15 bp) were localized in the target genomic region (Additional file 5). The 236 bp deletion observed in the P3/P6 amplicon from T2–10 and T2–31, as well as in 4 other plants in the heteroplasmic condition, most likely results from the recombination of two 11 bp direct repeats (TGGGCGAGAGG, DR19a in the middle of the *orf125* coding sequence and DR19b in the intergenic region approximately 60 bp downstream) flanking the two TALEN2-binding sequences and the intervening site for *Fok*I cleavage (Fig. 3A and Additional file 3A), whereas the 1066 bp deletion observed in the P3/P7 amplicon from T1–6, T1–29, T1–49 and T2–14 arises from the recombination of two 12 bp long direct repats (GGACCTTTCCTC, DR14a approximately 700 bp upstream of *orf125* and DR14b at its 3′ end) flanking both TALEN1 and TALEN2 pairs and the respective sites subjected to the double strand break (Fig. 3B and Additional file 3B).

The two recombination events involving DR19 and DR14 resulted in the deletion of the second half or almost the entire *orf125* coding sequence (the latter was reduced to the first 210 bp or the last 17 bp, respectively). In both cases, a single copy of each DR pair was still present in the recombined sequence (Fig. 3C, Additional file 3A, B). After sequencing the P3/P11 amplicon shown in T2–12, a deletion of 4288 bp between nucleotides 1884 and 6173 was confirmed, but its origin was unclear (Fig. 3D and Additional file 3C).

Table 1 summarizes the editing events obtained with the mitoTALEN approach. Overall, 3 out of 15 plants were edited using TALEN1 sequences, and 8 out of 14 were edited with TALEN2 sequences. Considering both the percentage of edited plants and that of homoplasmic plants, the overall efficiency was found to be higher at the T2 site than at the T1 site.

**Table 1:** Summary of editing events observed with TALEN1 and TALEN2 sequence pairs

Site	Plants (no.)				
	Analysed	Edited			
		Total	236 bp deletion	1066 bp deletion	4288 bp deletion
T1	15	3	–	3^a^	–
T2	14	8	6^b^	1	1

^a^Three heteroplasmic

^b^Four heteroplasmic, one of which also showed a 4 bp insertion

### MitoTALECD

**Fig. 4:**
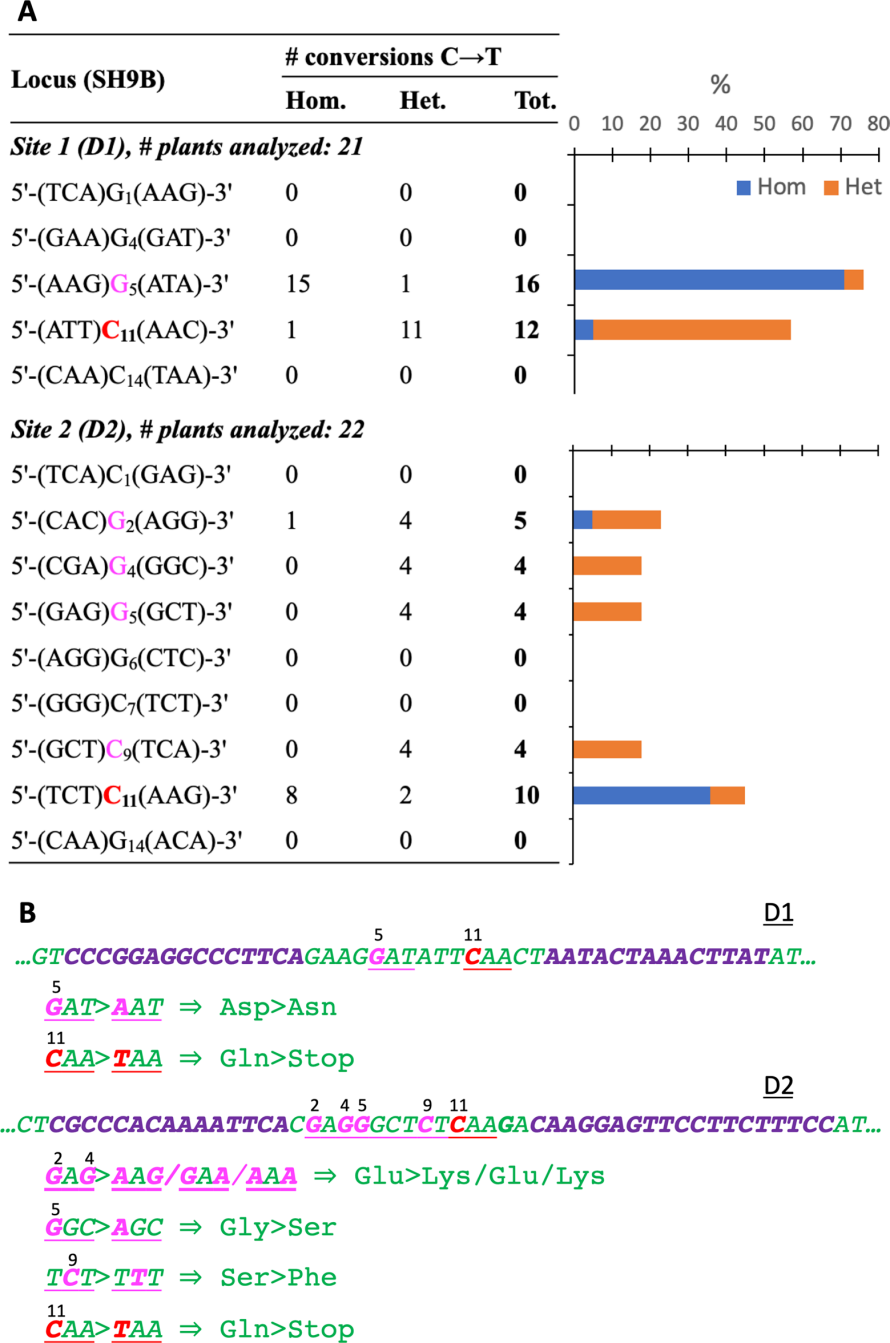
Base editing of *orf125* with TALECD1 (D1) and TALECD2 (D2) pairs. **A** Number and frequency of editing events in transgenic plants; **B** Nucleotide changes observed in the spacing window and expected changes in derived proteins. Modified C/Gs in the coding strand are numbered from the 5′ start of the spacing window. Nucleotide changes determining nonsense and missense mutations are indicated in red and magenta, while *orf125* and TALECD sequences are reproduced in green and violet, respectively. *Hom* homoplasmic, *Het* heteroplasmic

Thirty-four and 39 kanamycin-resistant shoots were obtained in the TALECD experiments, each based on 105 explants. After PCR confirmation, *nptII*^*+*^ plants were Sanger sequenced with primers P4/P5 (Additional file 2). The outcomes of the two experiments (D1, TALECD1, and D2, TALECD2) are shown in Fig. 4 and Additional file 6. With the TALECD1 pair, 16 and 12 of 21 plants (76 and 57%, respectively) were edited in the G in the fifth position of the target window (C_11_ in the complementary strand) and in C_11_, respectively. The G_5_→A substitution induced an Asp→Asn change, whereas the C_11_→T substitution resulted in the desired stop codon, producing a protein of 56 aa instead of 125. However, most edited plants were homoplasmic in the first case (15) but heteroplasmic in the second (11). Most plants showed some editing at both sites (Additional file 7A). The Cs in other positions were not edited.

Using the TALECD2 pair, five Cs/Gs in the target window were edited (G_2_, G_4_, G_5_, C_9_, C_11_), although at variable frequencies. C_11_ was changed to T in 10 out of 22 plants (45%), and the majority of them were homoplasmic for the mutation, resulting in a premature stop codon and shorter protein (88 aa). On the other hand, the editing frequency was lower overall at other sites, and most plants were heteroplasmic. A combination of amino acid changes was present in edited plants (Additional file 7B). Overall, 1.23 mutations per plant (0.41 homoplasmic) were obtained with the TALECD2 pair,compared to 1.33 (0.76 homoplasmic) with TALECD1, but the frequency of homoplasmic mutations in the target C was lower in the latter, suggesting greater efficiency and specificity of the TALECD2 pair.

### Stability of editing outcomes

The genotype of a sample of vegetative progenies (G1) derived by tuber propagation of first generation (G0) edited and unedited plants is reported in Table 2 and Additional file 8.

**Table 2:** Genotype of vegetatively propagated plants (G1) at the target loci. Induced mutations are in bold

Clone	Genotype G0^a^	Genotype G1^b^
SH9B (P3–P6 = 983 bp; P3–P7 = 1919 bp; P3–P11 = 5101 bp)^c^		
T1–6 (P3–P7)	**Het Del1066**	**Het Del1066**
T1–29 (P3–P7)	**Het Del1066**	**Het Del1066**
T1–49 (P3–P7)	**Het Del1066**	**Het Del1066** + **R**
T2–1 (P3–P6)	**Het Del236**	**Het Del236**
T2–3 (P3–P6)	WT	WT
T2–10 (P3–P6)	**Hom Del236**	**Hom Del236**
T2–12 (P3–P11)	**Hom Del4288**	**Hom Del4288**
T2–14 (P3–P7)	**Hom Del1066**	**Hom Del1066**
T2–15 (P3–P6)	WT	WT
T2–23 (P3–P6)	**Het Del236**	WT
T2–26 (P3–P6)	**Het Del236** + **Ins4**	WT (**Ins4**)
T2–28 (P3–P6)	WT	WT
T2–29 (P3–P6)	**Het Del236**	**Het Del236** + **R**
T2–30 (P3–P6)	WT	WT
T2–31 (P3–P6)	**Hom Del236**	**Hom Del236**
SH9B (G_5_AT; C_11_AA)^d^		
D1–48	**A**AT; **C/T**AA	AAT; TAA
D1–51	**A**AT; CAA	**A**AT; CAA
D1–69	**A**AT; **C/T**AA	**A**AT; **C/T**AA
D1–70	**A**AT; CAA	**A**AT; **C/T**AA
D1–84	**A**AT; **T**AA	**A**AT; **T**AA
D1–90	**A**AT; **C/T**AA	**A**AT; **C**AA
D1–93	**A**AT; **C/T**AA	**A**AT; **C/T**AA
D1–113	**G/A**AT; CAA	**G/A**AT; **C/T**AA
SH9B (G_2_AG_4_; G_5_GC; TC_9_T; C_11_AA)^d^		
D2–9	GAG; GGC; TCT; CAA	GAG; GGC; TCT; CAA
D2–23	GAG; GGC; TCT; **T**AA	GAG; GGC; TCT; **T**AA
D2–27	GAG; GGC; TCT; **C/T**AA	GAG; GGC; TCT; **C/T**AA
D2–32	GAG; GGC; TCT; **C/T**AA	GAG; GGC; TCT; CAA
D2–33	GAG; GGC; TCT; CAA	GAG; GGC; TCT; CAA
D2–34	GAG; GGC; TCT; **T**AA	GAG; GGC; TCT; **T**AA
D2–38	GAG; GGC; TCT; CAA	GAG; GGC; TCT; CAA

^a^G0, first-generation plants after regeneration

^b^G1, Clones derived by tuber propagation of G0 plants, except for T2–1, T2–29, D1–70 and D1–113, which analysed progenies derived by several cycles of axillary bud propagation in vitro

^c^T1 and T2 plants were analysed by PCR with primer pairs P3-P6, P3-P7 or P3-P11 (see Additional file 2: Fig. S2). The size of the PCR fragments in SH9B is indicated. The mutated genotype in the G0 and G1 generations is represented by the size (bp) of the deletion in homoplasmic (Hom) or heteroplasmic (Het) conditions. Ins4 in T2-26 stands for a 4 bp insertion into the largest WT amplicon, and R in T1-49 and T2-29 stands for novel bands putatively derived from mtDNA rearrangements

^d^D1 and D2 plants were analysed by sequencing the fragment obtained by PCR with primers P4 and P5. The genotype of the selected codons in the two target windows is reported. Small numbers indicate the C/G positions in the sense strand

As far as mitoTALEN-derived plants are concerned, all the unedited plants (T2–3, T2–15, T2–28, T2–30) gave origin to unedited progenies, while the homoplasmic plants (T2–10, T2–12, T2–14, T2–31) confirmed the original deletion. However, the original heteroplasmic plants (T1–6, T1–29, T1–49, T2–1, T2–23. T2–26, T2–29) either showed only the wild-type amplicon or confirmed heteroplasmy, but with an overall predominance of the WT amplicon. In two cases (T1–49 and T2–29), novel additional products appeared after PCR.

In the case of base-editing experiments, unedited plants (D2–9, D2–33 and D2–38) always gave rise to unedited progenies. In edited plants, the original homoplasmic substitutions were always confirmed, while the heteroplasmic substitutions remained unchanged or evolved towards either wild-type or mutated homoplasmic conditions. In only a couple of plants (D1–70 and D1–113), the original unedited CAA codons became heteroplasmic (C/TAA) in the offspring.

## Discussion

Plants with edited mitochondrial genomes were obtained at high frequency in the present study using either the mitoTALEN or the mitoTALECD approaches. In both cases, two sites were identified to target the TALE sequence pairs, and total or partial physical deletion of the target gene or insertion of a stop codon was achieved. Edited plants showed no obvious alteration in plant growth or other vegetative traits. Preliminary data indicated reversion to male fertility, but their phenotypic characterization is under way (manuscript in preparation). In this study, we focused on detailed molecular characterization and stability of editing events and induced chondriome mutations.

Various outcomes have been obtained in the experiments with the nuclease. Due to the frequent occurrence of short direct repeats in the target region, two of them, 11 and 12 bp long, were involved in ectopic homologous recombination to produce relatively short deletions (236 and 1066 bp, respectively) comprising the target *orf125* (putative) gene to varying degrees. This is somehow in contrast not only with genome editing approaches in the nuclear genome, where short indels are usually obtained after repairing the DSB *via* the NHEJ pathway [32] but also with previous reports on editing of the mitochondrial genome based on the use of mitoTALEN. In fact, unlike mammalian mitochondrial genomes, where degradation of edited molecules is generally observed [33], in plant chondriomes, repair of the induced DSB was previously achieved by homologous recombination of distant repeat pairs, with one copy close to the DSB and one elsewhere in the genome, generally leading to large deletions [15, 24–27].

In this study, the proximity of the target *orf* to the essential single-copy *nad4* gene (only 200 bp downstream) and *orf247* with unknown function (approximately 4 kbp upstream) likely prevented large rearrangements from occurring in regenerated plants. Indeed, the presence of the two flanking genes was preserved in all edited events. Although some plants were heteroplasmic, in other cases, wild-type molecules were not visible, suggesting an early and efficient occurrence of editing before regeneration. A particular case of heteroplasmy, leading to a double mutation, was observed in plant T2-26, where in addition to the 236 bp deletion, a 4 bp insertion, likely derived by a rare error-prone illegitimate microhomology-mediated mechanism [34], has also been observed for some molecules.

While large repeats (> 500 bp) in mitochondrial genomes are usually involved in frequent and reversible intramolecular homologous recombination events, leading to the emergence of multiple circular subgenomes, medium- to short-sized repeats (< 500 bp) are responsible for rare irreversible events involving either ectopic homologous or illegitimate microhomology-mediated recombination [34]. The need to repair the induced DSB favors the recombination of such repeats present either in the surroundings, as in our case, or one next to the DSB and another somewhere else in the genome, as shown in other species.

A regenerated plant (T2–12) showed a PCR fragment indicative of a major deletion of approximately 4300 bp, completely deleting the target site, but we have thus far been unable to find the repeats likely involved in the recombination. The same fragment, however, was barely visible in the original SH9B genotype, suggesting that the induced DSB and subsequent repair led to the amplification of an already present substoichiometric molecule. Such a specific change in copy number, a phenomenon known as substoichiometric shift, was also observed in rice plants edited by mitoTALEN in *orf312* [26]. In other studies, it was found responsible for modifications in chondriome organization and the appearance of new traits after changes in the nuclear background and/or stress derived from in vitro culture [34].

We also produced edited plants using the mitoTALECD approach that exploits the ability of the DddA deaminase derived from *Burholderia cenocepacia* to convert Cs to Ts in double-stranded DNA [20], paving the way for organellar base editing both in mammals and plants [35]. In both systems, it has been reported that the efficiency of C→T conversions is affected by several factors: presence of an UGI (uracyl glycosylase inhibitor) molecule in the construct, nucleotide context, with 5′-TC-3′ being largely favoured, length of the spacing region between TALEs and position of Cs therein, split type and orientation of deaminase components [20, 36, 37]. We observed variable efficiencies in different contexts, considering the overall changes and the frequencies of homoplasmic versus heteroplasmic ones. Using two TALE pairs, target C (eleventh from the start of the spacing region) was changed to T with a similar overall frequency, but the frequency of homoplasmy was significantly different. Considering that the position of the C in the two sites was the same and that in both cases, a T preceded the C, other factors were likely involved in editing efficiency.

Overall, out of a total of 55 editing events, 47 involved a 5′-TC-3′ context and 8 a 5′-CC-3′ context, confirming greater relaxation of the system in plant cells than in mammals [20, 22, 23, 28, 29, 37]. Only alternative 5′-AC-3′ and 5′-GC-3′ were occasionally observed in recent reports on plants, and changes in 5′-CC-3′ contexts were shown only if a T preceded the two Cs, giving rise to consecutive double modifications.

Consistent with other reports, additional mutations were observed for some further Cs present in both strands of the spacing region (bystander edits), but reasons for variations in overall frequency and/or degree of homoplasmy, such as in C_11_ and G_5_ (C_11_ on the opposite strand) in the first target site (D1), were not always evident. The presence of bystander edits leading to missense mutations, however, can be exploited to generate further variations useful for understanding the role of specific sequences in organellar biology. On the other hand, attempts can be made to reduce the frequency of bystander changes by sliding the TALE recognition targets, using different sized target windows, or optimizing the sequences linking the CD subunit to TALE [22, 23]. No other changes in the *orf125* gene sequence outside the target window were observed in the present study (Mendeley Data Repository, doi: 10.17632/s82t78sk5m.1). An overall low frequency of off-target effects has been, however, reported in other systems [20, 22, 23, 29, 38].

In our hands, both mitoTALEN and mitoTALECD approaches have proven effective methods to generate novel variability in the mitochondrial genome, leading to knock-out of the target sequence. Some differences in the efficiency of the TALE sequences tested could be observed, suggesting that more combinations should be tested. The accuracy of the nuclease-based method was improved by the fortunate presence of several small direct repeats in the target region, favouring small-size precise deletions over situations where the recombination of distant repeats can lead to large genome rearrangements and gene losses. Therefore, regions rich in small direct repeats can be conveniently used in targeted biotechnological approaches aimed at mitochondrial DNA editing. On the other hand, the deaminase-based approach, allowing a more targeted approach, permits not only gene knock-outs following the induction of nonsense mutations but also modifications of the gene product due to missense mutations. Novel DddA variants have recently been developed to expand the efficiency and scope of base editing in organellar genomes [36–39].

The homoplasmic mutations detected in the regenerated potato plants were always confirmed in the vegetative progenies after tuber propagation, indicating the stability of the mutated genotype, induced either by the mitoTALEN or mitoTALECD approach. Similarly, the genotype of unedited plants was also generally confirmed, suggesting the absence of “late” editing, possibly due to reduced nuclease or deaminase expression in some transgenic plants. On the other hand, heteroplasmy was generally found to be a quite unstable condition, especially in plants showing mitoTALEN-induced deletion. The appearance of novel bands in the progenies of some mitoTALEN-derived heteroplasmic plants suggests further rearrangements involving edited and/or wild-type mtDNA molecules.

For regulatory and public acceptance issues, especially in vegetatively propagated species such as potato, where it is difficult to eliminate nuclear-inducing constructs, it would be worth developing transient delivery methods to induce TALEN- or TALECD-based editing of organellar genomes. This seems feasible because in several species, such as Brassica, Arabidopsis and rice, mutations induced in plant organelles by both approaches were shown to be stably inherited by seed progenies even after segregation of the inducing nuclear genes [15, 22, 23, 26, 27]. In addition, DNA-free base editing of the plastome was achieved by delivering cytidine deaminase mRNA to lettuce protoplasts [29].

## Conclusions

We developed two TALE-based approaches to edit the mitochondrial genomes in potato. Both allowed us to obtain plants with precisely modified mitochondrial genomes at high frequency. The use of the same plant genotype and mtDNA region allowed us to compare the two methods for efficiency, accuracy, type of modifications induced and stability after vegetative propagation. The possibility to induce directed (short) deletions or point mutations in the potato chondriome will allow to study and exploit also in this species uncharacterized open reading frames and their interactions with nuclear genes.

## Methods

### Target settings and vector construction

Two target bases of mitoTALECDs were selected as the sites allowing “TAA” stop codons to be caused by “CAA”s editing in *orf125*. The target base Cs was set at the 11th base of the 15 bp target windows between the recognized sequences by the pairs of which the 1397 N halves were set as upstream ones. The effective recognition sequences are “tCCCGGAGGCCCTTCA”, “tATAAGTTTAGTATT”, “tCGCCCACAAAATTCA”, and “tGGAAAGAAGGAACTCCTTG”, of which t indicates the base recognized by the N-terminal domains of the TALE repeats. Sequences recognized by mitoTALENs were selected as close to the above TALECD-recognizing sequences from the lists using the website TALEN Targeter (https://tale-nt.cac.cornell.edu/node/add/talen-old) as a query sequence of *orf125*. The recognized sequences are “tTCAGAAGGAT”, “tTAGTCAAATATAAGTT”, “tCGCCCACAAAATTCACGAGG”, and “tGGAAAGAAGGAACT”.

The binary vectors for dual expression of pairs of mitoTALENs and mitoTALECDs were assembled according to previous reports [15, 22, 23]. In brief, DNA-recognizing repeats, platinum TALE repeats, were assembled by using a Platinum Gate TALEN kit (Kit #1000000043, Addgene [40]), and the latter part for assembling two pairs of TALE-Ns or TALE-CDs was assembled by Multisite Gateway technology (Invitrogen) with vectors provided by Addgene [15, 22, 23].

### Plant transformation

The four binary vectors pTALEN1/2 (mitoTALEN) and pTALECD1/2 (mitoTALECD) were separately electroporated into *Agrobacterium tumefaciens* strain EHA105.

Leaf explants of *Solanum tuberosum* (+) *S. commersonii* somatic hybrid SH9B [31] were excised from sterile plants grown in vitro and used in individual transformation experiments (approximately 100 explants per transformation) with the four strains of *A. tumefaciens* previously prepared, according to the protocol by [41]. The shoots regenerated on selective regeneration medium (kanamycin 100 mg/L) were transferred to rooting selective medium (kanamycin 25 mg/L), and only plants with well-developed roots were subjected to downstream in-depth analyses.

### PCR screening and DNA sequencing

Total DNA was extracted from the leaves of putatively transgenic plants using the GeneJET Plant Genomic DNA Purification Kit (Thermo Scientific, Waltham, Massachusetts, USA) according to the manufacturer’s instructions. PCR was carried out to detect the selectable marker gene *NPTII* using primers NPTIIFor (5′-GATGGATTGCACGCAGGTTC-3′; Tm: 64 °C) and NPTIIRev (5′- GATGTTTCGCTTGGTGGTCG-3′; Tm: 64 °C) with Phire Hot Start II DNA polymerase (Thermo Scientific, Waltham, Massachusetts, USA) according to the manufacturer’s instructions.

The transgenic plants were further analysed by PCR with specific primers (Additional file 2) for the mitochondrial region investigated with the two TALEN pairs (TALEN1-L/R and TALEN2-L/R) and the two TALECD pairs (TALECD1-L/R and TALECD2-L/R) using Phusion or Phire Hot Start II (Thermo Scientific, Waltham, Massachusetts, USA) (DNA polymerases, according to the manufacturer’s instructions). All primers were purchased from Eurofins (Eurofins Scientific, Luxemburg). PCR products were subjected to gel electrophoresis to detect the expected amplicon size (Additional file 2), and digital images were acquired with the Gel Doc XR Imaging System (Bio-Rad, Hercules, California, USA).

All PCR products from the TALECD1 and TALECD2 transgenic plants and those from the TALEN1 and TALEN2 transgenic plants showing differences in expected size were purified with the GeneJet Gel extraction kit (Thermo Scientific, Waltham, Massachusetts, USA) and Sanger sequenced (Eurofins Scientific, Luxemburg). Sequences were analysed with CLC Main Workbench 8 (Qiagen, Hilden, Germany) and deposited in Mendeley Data repository (doi: 10.17632/s82t78sk5m.1). Sequences derived from regenerated plants putatively showing a deletion were aligned with those of SH9B using the Stretcher (EMBOSS) EMBL-EBI tool [42].

### High-resolution fragment analysis

PCR amplification was carried out in TALEN1 and TALEN2 transgenic plants with a 5′ FAM-modified P4 primer (incorporating the fluorescent 6-fluorescein amidite moiety) and P5 (Additional file 2). Each PCR product was subsequently diluted 1:200 in water, and 1 µL was mixed with 13.7 µL of Hi-Di Formamide (Thermo Scientific, Waltham, Massachusetts, USA) and 0.3 µL of Gene Scan 600 Liz v 2.0 (Thermo Scientific, Waltham, Massachusetts, USA). The denatured samples were then run on a SeqStudio Genetic Analyser (Thermo Scientific, Waltham, Massachusetts, USA), and the results were analysed with Gene Mapper 5 (Thermo Scientific, Waltham, Massachusetts, USA).

### Repeat identification

Perfect direct repeats in the target region were identified using the web tool accessible at https://www.novoprolabs.com/tools/repeats-sequences-finder and setting 10 bp as the minimum repeat length.

### Stability of editing outcomes

The regenerated plants were brought to maturity and propagated vegetatively from tubers. The progenies from a sample of unedited and edited plants were analysed again by PCR analyses with appropriate primers (T1 and T2 mitoTALEN-derived plants) or by sequencing (D1 and D2 mitoTALECD-derived plants). In a few cases where tubers were not available, the analyses were repeated in progenies derived from several cycles of axillary bud proliferation in vitro.

### Supplementary Information

**Additional file 1.** Vectors and strategies employed for mtDNA editing through the mitoTALEN and mitoTALECD approaches.

**Additional file 2.** Position and sequence of primers used for analyses of the *nad4*-*orf247* genomic region in SH9B and edited plants.

**Additional file 3.** Sequence alignment of PCR products obtained after amplification with various primer pairs. **A** P6 and P3 primer pair; **B** P7 and P3 primer pair; **C** P11 and P3 primer pair.

**Additional file 4.** Amplification of *nad4* (first two exons) and *orf247* with primer pairs P1–P2 and P10–P11, respectively.

**Additional file 5.** Sequence and position of direct repeats (DR) in the SH9B genomic region comprised between primers P11 (*orf247*) and P3 (*nad4 exon 1*).

**Additional file 6.** Representative examples of sequences in the spacing window after base editing of *orf125* with TALECD1 (D1) and TALECD2 (D2) pairs.

**Additional file 7.** Codon changes in regenerated plants after base editing of *orf125 *with TALECD1 and TALECD2 pairs.

**Additional file 8.** PCR amplification with the indicated primer pairs of a sample of vegetatively propagated plants derived from mitoTALEN experiments. C^−^, negative control without DNA**.**

## Data Availability

All data generated or analysed during this study are included in this published article and its Additional files.
