# Supplementary figures and images for: Mitochondrial DNA editing in potato through mitoTALEN and mitoTALECD: molecular characterization and stability of editing events
Source: Plant Methods. 2024 Jan 5;20:4. doi: 10.1186/s13007-023-01124-9 (PMC10768376; doi:10.1186/s13007-023-01124-9)

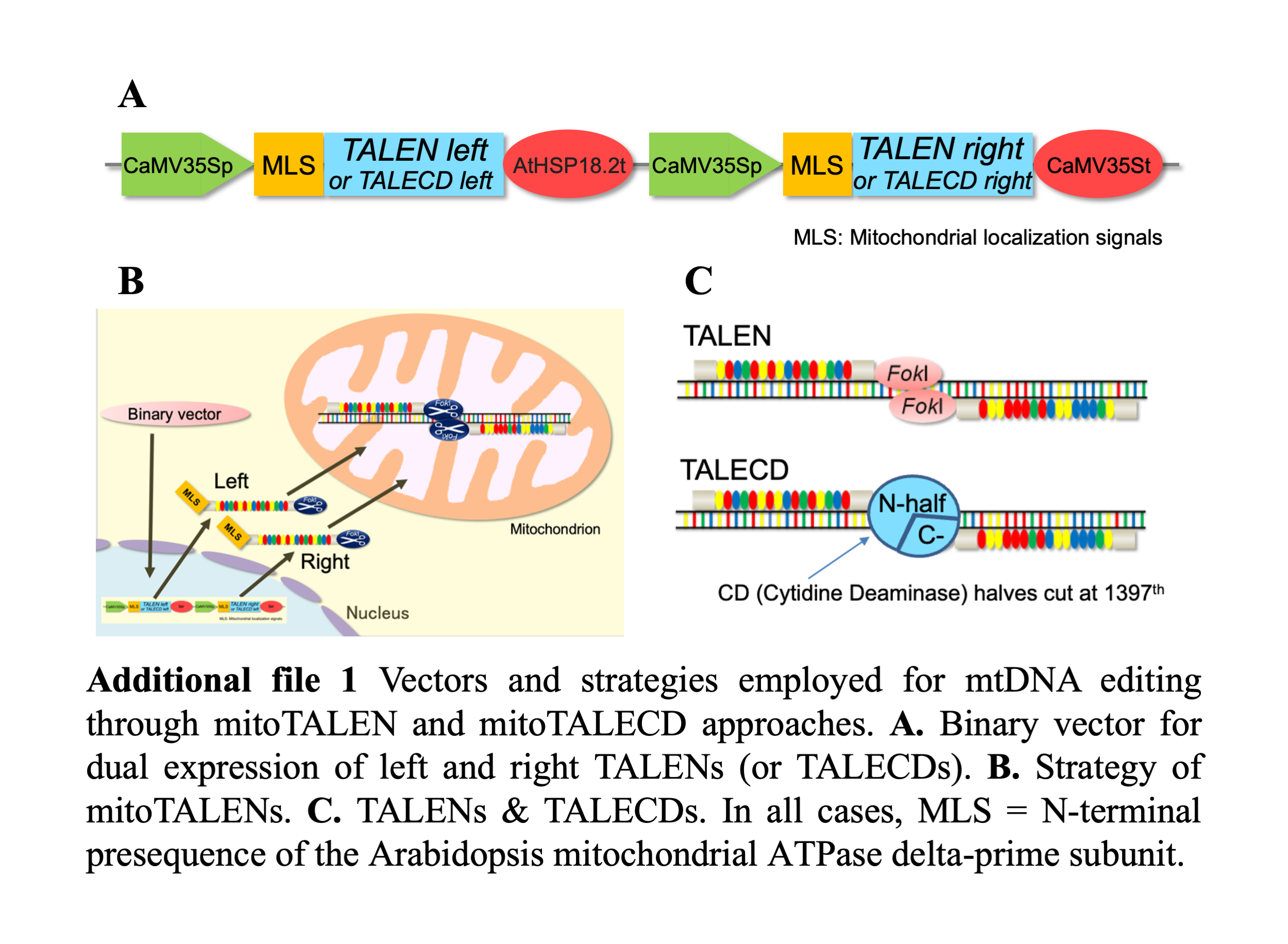

Supplement: Supplementary file 1 — Additional file 1. Vectors and strategies employed for mtDNA editing through the mitoTALEN and mitoTALECD approaches. [file 13007_2023_1124_MOESM1_ESM.tiff]

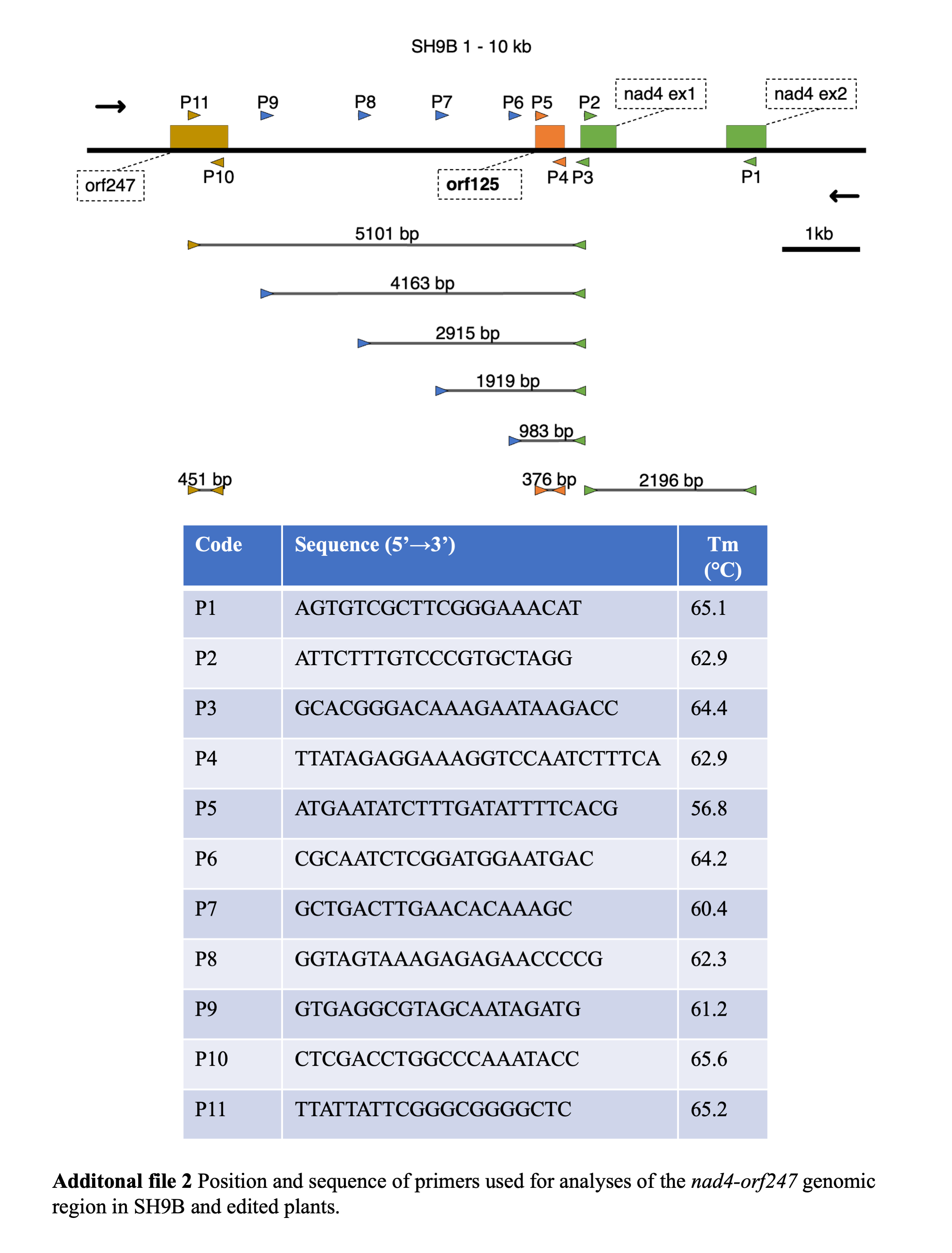

Supplement: Supplementary file 2 — Additional file 2. Position and sequence of primers used for analyses of the nad4-orf247 genomic region in SH9B and edited plants. [file 13007_2023_1124_MOESM2_ESM.tiff]

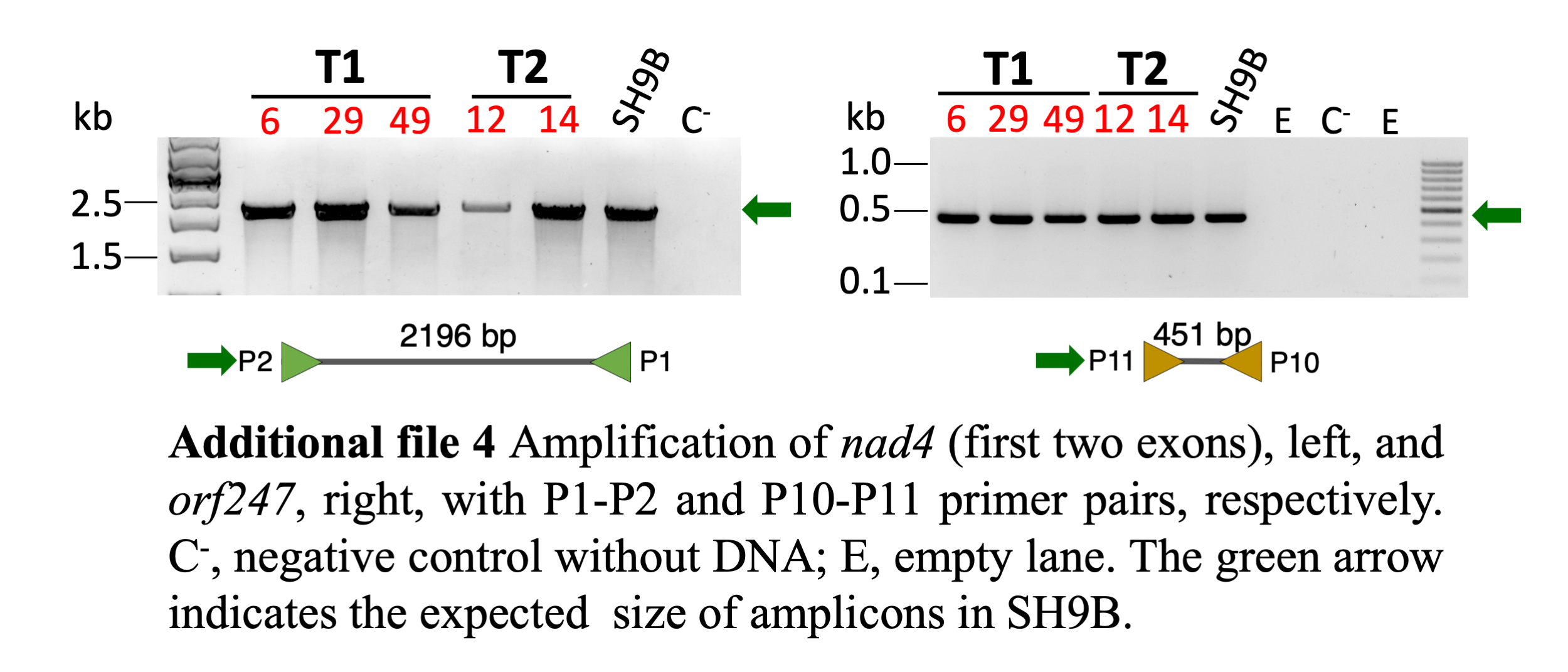

Supplement: Supplementary file 4 — Additional file 4. Amplification of nad4 (first two exons) and orf247 with primer pairs P1–P2 and P10–P11, respectively. [file 13007_2023_1124_MOESM4_ESM.tiff]

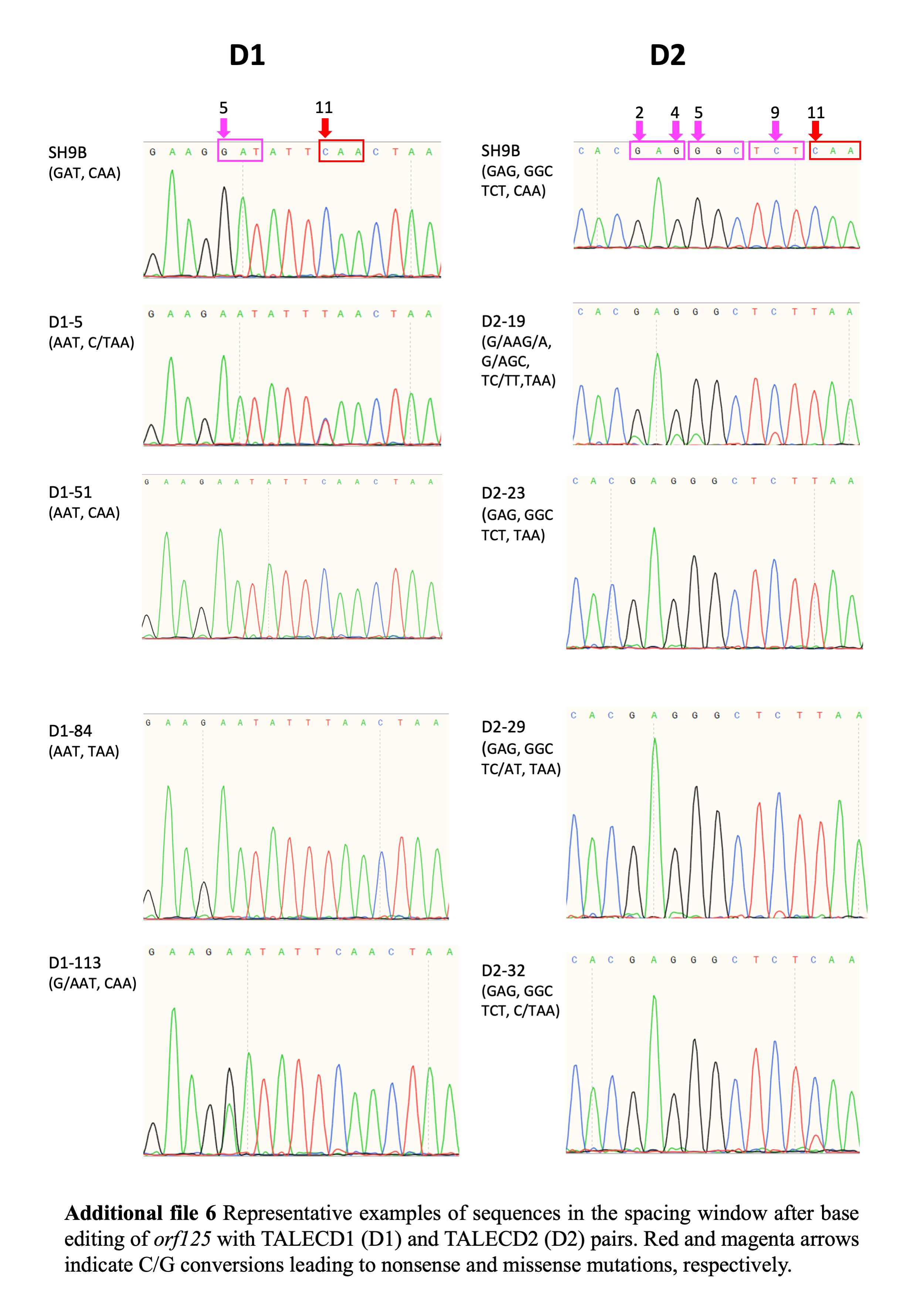

Supplement: Supplementary file 6 — Additional file 6. Representative examples of sequences in the spacing window after base editing of orf125 with TALECD1 (D1) and TALECD2 (D2) pairs. [file 13007_2023_1124_MOESM6_ESM.tiff]

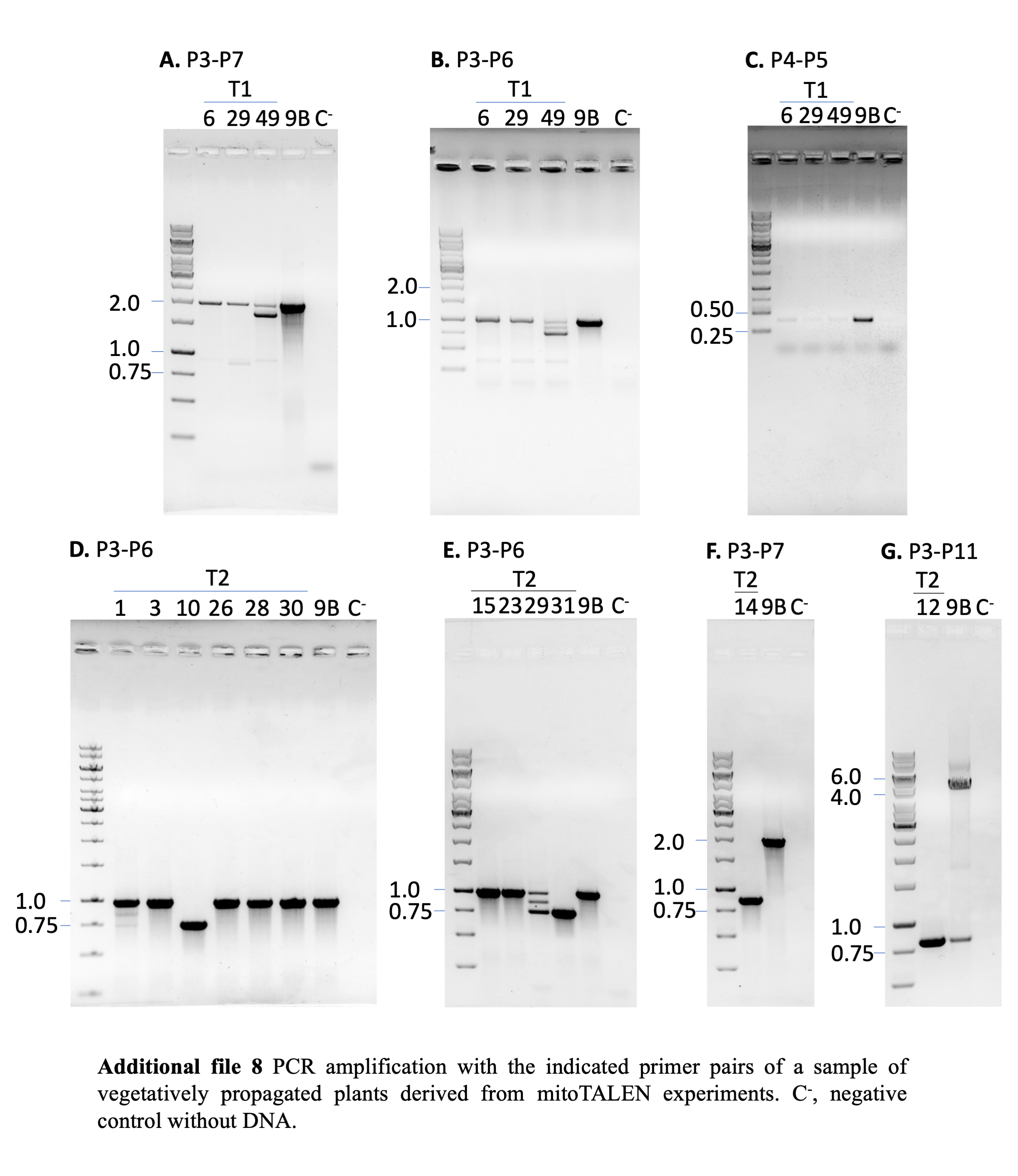

Supplement: Supplementary file 8 — Additional file 8. PCR amplification with the indicated primer pairs of a sample of vegetatively propagated plants derived from mitoTALEN experiments. C−, negative control without DNA. [file 13007_2023_1124_MOESM8_ESM.tiff]
