# Supplementary material for: Mitochondrial DNA editing in potato through mitoTALEN and mitoTALECD: molecular characterization and stability of editing events
Source: Plant Methods. 2024 Jan 5;20:4. doi: 10.1186/s13007-023-01124-9 (PMC10768376; doi:10.1186/s13007-023-01124-9)
Supplement: Supplementary file 3 — Additional file 3. Sequence alignment of PCR products obtained after amplification with various primer pairs. A P6 and P3 primer pair; B P7 and P3 primer pair; C P11 and P3 primer pair. [file 13007_2023_1124_MOESM3_ESM.pdf]

A. P6-P3

```
P6-1 CGCAATCTCGGATGGAATGACCTTTTGCTCCTTGATGAAACTCACATGAT     50
                     ||||||||||||||||||||||||||||||||||
   1                 ATGACCTTTTGCTCCTTGATGAAACTCACATGAT     34

  51 TCATCTTTTTCATAACTAGTTACTTGAGATTGCTCTTCGTAAAGTTCTGG    100
     ||||||||||||||||||||||||||||||||||||||||||||||||||
  35 TCATCTTTTTCATAACTAGTTACTTGAGATTGCTCTTCGTAAAGTTCTGG     84

 101 TTGGACATAATGAGTGTAACGAAAAGAAAGAAAGACTAGAAGACATAGGG    150
     ||||||||||||||||||||||||||||||||||||||||||||||||||
  85 TTGGACATAATGAGTGTAACGAAAAGAAAGAAAGACTAGAAGACATAGGG    134

 151 AATTTCAAGTAATACGTATTCGTTTATCAACTCATTTCGTCTCACTACGT    200
     ||||||||||||||||||||||||||||||||||||||||||||||||||
 135 AATTTCAAGTAATACGTATTCGTTTATCAACTCATTTCGTCTCACTACGT    184

 201 ATAGGGAATAGGGAAAAGCCTTGCCTTATTCAAGAGTACCAGGAAGGAGA    250
     ||||||||||||||||||||||||||||||||||||||||||||||||||
 185 ATAGGGAATAGGGAAAAGCCTTGCCTTATTCAAGAGTACCAGGAAGGAGA    234

 251 AGAATAAGCAGAATTGCTTAAGTAAGGTAGTCGCTTCTTTTTTTCGGTAT    300
     ||||||||||||||||||||||||||||||||||||||||||||||||||
 235 AGAATAAGCAGAATTGCTTAAGTAAGGTAGTCGCTTCTTTTTTTCGGTAT    284

 301 ACCGCTCTGCGAGCAGAGCGAATTAACATAAATCTTTCCGAATGAATATC    350
     ||||||||||||||||||||||||||||||||||||||||||||||||||
 285 ACCGCTCTGCGAGCAGAGCGAATTAACATAAATCTTTCCGAATGAATATC    334

 351 TTTGATATTTTCACGATTGCCTATTCAATGATTTTTTTTGTCTCTTTCAC    400
     ||||||||||||||||||||||||||||||||||||||||||||||||||
 335 TTTGATATTTTCACGATTGCCTATTCAATGATTTTTTTTGTCTCTTTCAC    384

 401 TTTTTTCGTAGCCAGACACAAACTTTCTTCCTATTTATCCTTTAATGACC    450
     ||||||||||||||||||||||||||||||||||||||||||||||||||
 385 TTTTTTCGTAGCCAGACACAAACTTTCTTCCTATTTATCCTTTAATGACC    434

 451 TTCTGACTAGAGAAGAATACCTTCGGCTGCTTGTCCCGGAGGCCCTTCAG    500
     ||||||||||||||||||||||||||||||||||||||||||||||||||
 435 TTCTGACTAGAGAAGAATACCTTCGGCTGCTTGTCCCGGAGGCCCTTCAG    484

 501 AAGGATATTCAACTAATACTAAACTTATATTTGACTAACTTGGGCGAGAG    550
     ||||||||||||||||||||||||||||||||||||||||||||||||||
 485 AAGGATATTCAACTAATACTAAACTTATATTTGACTAACTTGGGCGAGAG    534

 551 GATGCCCCAAGGGCACTCTTGGCAAGAGCTCGCCCACAAAATTCACGAGG    600
     |
 535 G-------------------------------------------------    535

 601 GCTCTCAAGACAAGGAGTTCCTTCTTTCCATTATATCTGACATTGTTTCT    650

 536 --------------------------------------------------    535

 651 AATGGAATGTCAAGTGAATGGGTCTTGATGGCTTTGCAAATCCTGAAAGA    700

 536 --------------------------------------------------    535

 701 TTGGACCTTTCCTCTATAATAGAAGGATATTTCAAGTATGGAAGAAATCT    750

 536 --------------------------------------------------    535

 751 TATTTTTTAGCCTCATTACTTCCCACTGGGCGAGAGGTGCACCTAACCCA    800
```

```
                                                  |||||||||||||
536 -------------------------------------TGCACCTAACCCA    548

801 CCTACCCACTCATCAATCACGGTGTTCAGCTGACTTGCACTGATAGACCC    850
    ||||||||||||||||||||||||||||||||||||||||||||||||||
549 CCTACCCACTCATCAATCACGGTGTTCAGCTGACTTGCACTGATAGACCC    598

851 CTATTGTATTGGAATTAGGCGCCCATCTTTTTACTGTTGTCAATGAATCT    900
    ||||||||||||||||||||||||||||||||||||||||||||||||||
599 CTATTGTATTGGAATTAGGCGCCCATCTTTTTACTGTTGTCAATGAATCT    648

901 CTTCCATGTTCGATTCTACTCTATGTTAGAACATTTCTGTGAATGCTATT    950
    ||||||||||||||||||||||||||||||||||||||||||||||||||
649 CTTCCATGTTCGATTCTACTCTATGTTAGAACATTTCTGTGAATGCTATT    698

951 CTGATCTAAGTGGTCTTATTCTTTGTCCCGTGC    983-P3
    ||||||||||||||||||
699 CTGATCTAAGTGGTCTTA                   716
```

B. P7-P3

```
P7-1 GCTGACTTGAACACAAAGCCAATCTTCGAGAGGTTAGTGGATAACTGTAA    50
                    |||||||||||||||||||||||||||||||||||
   1                AAGCCAATCTTCGAGAGGTTAGTGGATAACTGTAA    35

  51 CCAGTTCGTCCTTCTTCGGAGAAATCGGGGAGCGGGCTTAAGGCTAGGGC   100
     ||||||||||||||||||||||||||||||||||||||||||||||||||
  36 CCAGTTCGTCCTTCTTCGGAGAAATCGGGGAGCGGGCTTAAGGCTAGGGC    85

 101 CCCTCCGCGCATCAGAAATCCTTGCTTACGATCTGGAGAGAAGGATCTTT   150
     ||||||||||||||||||||||||||||||||||||||||||||||||||
  86 CCCTCCGCGCATCAGAAATCCTTGCTTACGATCTGGAGAGAAGGATCTTT   135

 151 TCCAAGAAAGACCTATTGCCCTTATTACTCCAAGAGATTCAAAGCGGCTT   200
     ||||||||||||||||||||||||||||||||||||||||||||||||||
 136 TCCAAGAAAGACCTATTGCCCTTATTACTCCAAGAGATTCAAAGCGGCTT   185

 201 TAGATTAGTTGATTAGACAATTCCTACTGCTGTGCTAAAGGCTAGAGAGG   250
     ||||||||||||||||||||||||||||||||||||||||||||||||||
 186 TAGATTAGTTGATTAGACAATTCCTACTGCTGTGCTAAAGGCTAGAGAGG   235

 251 CAAGAAGAACATCTTACTCAACAAGAGTATCGGAAGAGATGCGCCTTCGG   300
     ||||||||||||||||||||||||||||||||||||||||||||||||||
 236 CAAGAAGAACATCTTACTCAACAAGAGTATCGGAAGAGATGCGCCTTCGG   285

 301 GACTTCCCTTTTAGCGTGGGAGTAGAGAAGGAAAAGGCGAAGCTGAGTCA   350
     ||||||||||||||||||||||||||||||||||||||||||||||||||
 286 GACTTCCCTTTTAGCGTGGGAGTAGAGAAGGAAAAGGCGAAGCTGAGTCA   335

 351 TATAATAAGTAAGGGAACAAAGTGAGCTATAAGGCGATTGAATTCATTTC   400
     ||||||||||||||||||||||||||||||||||||||||||||||||||
 336 TATAATAAGTAAGGGAACAAAGTGAGCTATAAGGCGATTGAATTCATTTC   385

 401 AGTTTAGTTGATATGAAAGCTAGCAGAGCTCATATCGCATTTGAAAGCAA   450
     ||||||||||||||||||||||||||||||||||||||||||||||||||
 386 AGTTTAGTTGATATGAAAGCTAGCAGAGCTCATATCGCATTTGAAAGCAA   435

 451 GTGAGATATCACTATTGATTGATTGATTGATAGAAAGCCCTATTGTAGAC   500
     ||||||||||||||||||||||||||||||||||||||||||||||||||
 436 GTGAGATATCACTATTGATTGATTGATTGATAGAAAGCCCTATTGTAGAC   485

 501 TAATAGCTCTAGCTTTCGATTTGTTAGGAAGAGCTATTATTAATAGTAAT   550
```

```
    ||||||||||||||||||||||||||||||||||||||||||||||||||
486 TAATAGCTCTAGCTTTCGATTTGTTAGGAAGAGCTATTATTAATAGTAAT    535

551 TTCCCGCTTCAAGCAAAGTCAAGGACCTTTCCTCATTGGCCTTCGGCTTC    600
    ||||||||||||||||||||||
536 TTCCCGCTTCAAGCAAAGTCAA----------------------------    557

601 AGCAAATTGATCTGGGACCTTGTTTCGGTATAAGGACCAAGCCTTCGAGA    650

558 --------------------------------------------------    557

651 GTCATCCTTCTTGCTACTTTGTTTTGTTAGAGGGGGTTCTTTAGACTCTC    700

558 --------------------------------------------------    557

701 GGAACAAGGCTGTTGGTTGTTTCTCAGTCAGGCATGTCTGTGCTGCGCCA    750

558 --------------------------------------------------    557

751 ACGGGGATGGAGGAGATGCCCCGTCAGGAGGTGTTATGCGCAGACCACTC    800

558 --------------------------------------------------    557

801 AATCTTTTGGAAACTGGGTCCGCTGGGGCGGGTTCTTTGGTTAGCACACT    850

558 --------------------------------------------------    557

851 TATCTGGGAGCTTTGTTTTTCTTCATGACTGCCGATCATAATTCACTTCG    900

558 --------------------------------------------------    557

901 CATCCATCAAGCTCTGAACCTTCATTTTGAAATCTTCGCAATCTCGGATG    950

558 --------------------------------------------------    557

951 GAATGACCTTTTGCTCCTTGATGAAACTCACATGATTCATCTTTTTCATA   1000

558 --------------------------------------------------    557

1001 ACTAGTTACTTGAGATTGCTCTTCGTAAAGTTCTGGTTGGACATAATGAG   1050

558 --------------------------------------------------    557

1051 TGTAACGAAAAGAAAGAAAGACTAGAAGACATAGGGAATTTCAAGTAATA   1100

558 --------------------------------------------------    557

1101 CGTATTCGTTTATCAACTCATTTCGTCTCACTACGTATAGGGAATAGGGA   1150

558 --------------------------------------------------    557

1151 AAAGCCTTGCCTTATTCAAGAGTACCAGGAAGGAGAAGAATAAGCAGAAT   1200

558 --------------------------------------------------    557

1201 TGCTTAAGTAAGGTAGTCGCTTCTTTTTTTCGGTATACCGCTCTGCGAGC   1250

558 --------------------------------------------------    557

1251 AGAGCGAATTAACATAAATCTTTCCGAATGAATATCTTTGATATTTTCAC   1300

558 --------------------------------------------------    557
```

```
1301 GATTGCCTATTCAATGATTTTTTTTGTCTCTTTCACTTTTTTCGTAGCCA   1350

 558 --------------------------------------------------    557

1351 GACACAAACTTTCTTCCTATTTATCCTTTAATGACCTTCTGACTAGAGAA   1400

 558 --------------------------------------------------    557

1401 GAATACCTTCGGCTGCTTGTCCCGGAGGCCCTTCAGAAGGATATTCAACT   1450

 558 --------------------------------------------------    557

1451 AATACTAAACTTATATTTGACTAACTTGGGCGAGAGGATGCCCCAAGGGC   1500

 558 --------------------------------------------------    557

1501 ACTCTTGGCAAGAGCTCGCCCACAAAATTCACGAGGGCTCTCAAGACAAG   1550

 558 --------------------------------------------------    557

1551 GAGTTCCTTCTTTCCATTATATCTGACATTGTTTCTAATGGAATGTCAAG   1600

 558 --------------------------------------------------    557

1601 TGAATGGGTCTTGATGGCTTTGCAAATCCTGAAAGATTGGACCTTTCCTC   1650
                                           ||||||||||||
 558 --------------------------------------GGACCTTTCCTC    569

1651 TATAATAGAAGGATATTTCAAGTATGGAAGAAATCTTATTTTTTAGCCTC   1700
     ||||||||||||||||||||||||||||||||||||||||||||||||||
 570 TATAATAGAAGGATATTTCAAGTATGGAAGAAATCTTATTTTTTAGCCTC    619

1701 ATTACTTCCCACTGGGCGAGAGGTGCACCTAACCCACCTACCCACTCATC   1750
     ||||||||||||||||||||||||||||||||||||||||||||||||||
 620 ATTACTTCCCACTGGGCGAGAGGTGCACCTAACCCACCTACCCACTCATC    669

1751 AATCACGGTGTTCAGCTGACTTGCACTGATAGACCCCTATTGTATTGGAA   1800
     ||||||||||||||||||||||||||||||||||||||||||||||||||
 670 AATCACGGTGTTCAGCTGACTTGCACTGATAGACCCCTATTGTATTGGAA    719

1801 TTAGGCGCCCATCTTTTTACTGTTGTCAATGAATCTCTTCCATGTTCGAT   1850
     ||||||||||||||||||||||||||||||||||||||||||||||||||
 720 TTAGGCGCCCATCTTTTTACTGTTGTCAATGAATCTCTTCCATGTTCGAT    769

1851 TCTACTCTATGTTAGAACATTTCTGTGAATGCTATTCTGATCTAAGTGGT   1900
     ||||||||||||||||||||||||||||||||||||||||||||||||||
 770 TCTACTCTATGTTAGAACATTTCTGTGAATGCTATTCTGATCTAAGTGGT    819

1901 CTTATTCTTTGTCCCGTGC    1919-P3
     |||||||
 820 CTTATTC                 826
```

C. P11-P3

```
P11-1 TTATTATTCGGGCGGGGCTCTTGAAGGCTTCCTTGCCGCGTGCGACGCGG      50
                                         |||||||||||
    1                                    GTGCGACGCGG      11

   51 GAAGGGGTTCCCCGTCTGGCCCTTTTTACCCTCCTGGGAGAGAAGCACAG     100
      ||||||||||||||||||||||||||||||||||||||||||||||||||
   12 GAAGGGGTTCCCCGTCTGGCCCTTTTTACCCTCCTGGGAGAGAAGCACAG      61
```

```
101 CCGCACATTCCCGAGCCCGAGGTTGCGCCCATTCCGCGCGCCGTTCTGGT    150
    ||||||||||||||||||||||||||||||||||||||||||||||||||
 62 CCGCACATTCCCGAGCCCGAGGTTGCGCCCATTCCGCGCGCCGTTCTGGT    111

151 TCCGGAATTAGAACGCCCTCTCCTTCCTGATATCCAAAGGAGAGCGGAGC    200
    ||||||||||||||||||||||||||||||||||||||||||||||||||
112 TCCGGAATTAGAACGCCCTCTCCTTCCTGATATCCAAAGGAGAGCGGAGC    161

201 TTCAACACCGGCTGTCCTTCTATTTTATCGGGAGGAACGAGCCCAGGCAC    250
    ||||||||||||||||||||||||||||||||||||||||||||||||||
162 TTCAACACCGGCTGTCCTTCTATTTTATCGGGAGGAACGAGCCCAGGCAC    211

251 CTCCCACTCTTTTTGGGTATCCTGGAGAAGCAGTTCCTCTTGGAAAAGAG    300
    ||||||||||||||||||||||||||||||||||||||||||||||||||
212 CTCCCACTCTTTTTGGGTATCCTGGAGAAGCAGTTCCTCTTGGAAAAGAG    261

301 GGTCGAGGCCGCATTGGTGGGGGATGGGTTTCCCCCCCAGATAGTTCTGG    350
    ||||||||||||||||||||||||||||||||||||| ||||||||||||
262 GGTCGAGGCCGCATTGGTGGGGGATGGGTTTCCCCCC-AGATAGTTCTGG    310

351 CTCGGAGGTCAGAAATCCGCAACCTCCTCTTTAACCACCCTACTAGGGGT    400
    ||||||||||||||||||||||||||||||||||||||||||||||||||
311 CTCGGAGGTCAGAAATCCGCAACCTCCTCTTTAACCACCCTACTAGGGGT    360

401 GGGGCGGCTCTCTCTGAGAATACCTTAAATAGGTATTTGGGCCAGGTCGA    450
    ||||||||||||||||||||||||||||||||||||||||||||||||||
361 GGGGCGGCTCTCTCTGAGAATACCTTAAATAGGTATTTGGGCCAGGTCGA    410

451 GCGGGATGGCACGCAGCAGAGCGTGCCCTATCTTAGGGTGCTTCGTGCTG    500
    ||||||||||||||||||||||||||||||||||||||||||||||||||
411 GCGGGATGGCACGCAGCAGAGCGTGCCCTATCTTAGGGTGCTTCGTGCTG    460

501 CCCGCTATTGGGTCTAAGTGGGTTCTCCCGCTTTTTCGCCTTTCAGAAGG    550
    ||||||||||||||||||||||||||||||||||||||||||||||||||
461 CCCGCTATTGGGTCTAAGTGGGTTCTCCCGCTTTTTCGCCTTTCAGAAGG    510

551 GTGGCCCACACACGGACAGGGGGAAAGGGGGGGAGACTAGGGAGTAATAT    600
    ||||||||||||||||||||||||||||||||||||||||
511 GTGGCCCACACACGGACAGGGGGAAAGGGGGGGAGACTAG----------    550

601 ACCATCTTTGACCATAATATACCAGGTGAAAAGAACCCATCTGGAATTGA    650

551 --------------------------------------------------    550

651 ATGAATTTTCTTTATATTCCAGATAAATGCTGCCAATTCATGCTTAAGAT    700

551 --------------------------------------------------    550

701 TCACAAAAATATTGTGGTCTTTAGCATATTTGCTAGCTACTTTATCGACT    750

551 --------------------------------------------------    550

751 GTATCGTCCACTTTTAAATAAAAACTACGGACGAAATTTTCTAATAAAGG    800

551 --------------------------------------------------    550

801 GGTACTTTTCCAAGTAGGCTCCCAAGACATACCTAATTCCAAGGGTATAT    850

551 --------------------------------------------------    550

851 CTTGAAGATGCGGTAAATAGATTGGTTGAAGTTCTTTAAAAGAGGTTTTA    900

551 --------------------------------------------------    550
```

```
901  ATCTCTCCTAAGACTTCTAGAACACTACCAATATCAGGGTGAGGCGTAGC    950

551  --------------------------------------------------    550

951  AATAGATGAAAAGGTTGCCTTATTCACTTTCTTAGTAACCAGAATCAGCT   1000

551  --------------------------------------------------    550

1001 TTGCCAAACCAAACCATGATAGGTACATTCGAACCAAAAGATCACCATGA   1050

551  --------------------------------------------------    550

1051 TCATACCTGGAGCGTATACTTTTACGTAACACTGCAGGTATGATTGTGGG   1100

551  --------------------------------------------------    550

1101 GATACCACACCTGGTCAGGGAAATTGGAACGGAAAGGGAATCACCTTTTT   1150

551  --------------------------------------------------    550

1151 GGTAATTACCCGCCTAATAACGCTGCAGGGATACTGCACACTCTTTTAAA   1200

551  --------------------------------------------------    550

1201 TACAAAGCAGTGAACAGAAAACCTGATTTCCGTTTCAAGGTCACTACCTT   1250

551  --------------------------------------------------    550

1251 TCTAACAAAGTCACAGCCCGCAAGGGCGATCTCTTTGGTCATGGAAGCAG   1300

551  --------------------------------------------------    550

1301 TGGCGATTAAAGGAACTCGAAAGAGGAGACCCTTTAACCGTCGGACTTGT   1350

551  --------------------------------------------------    550

1351 TCAAACAAGCCCCGCCAGGAGAACTTTGGTGTTCTTTCGATCGCACTGAA   1400

551  --------------------------------------------------    550

1401 GAGGATTTTAGTAATCATAATATTATTATATCCAAATCGTGTGAGCAGAA   1450

551  --------------------------------------------------    550

1451 AGGGGCAGCAAAGACTCTACTCTCCCTATCGGACTAAACTAGTCAGTCTC   1500

551  --------------------------------------------------    550

1501 GGGGGAAGGCGCCTGGGTTCCAACCAGGGGTTCACAAAGACACTAGCTCT   1550

551  --------------------------------------------------    550

1551 AGTATCACTACTAGAGGAAGCGCCAGAGTGAACTGGAGATTGTAATAAGA   1600

551  --------------------------------------------------    550

1601 TATTAGAATTTATGAGATAGGTACCATAAAACTGTCTGTTTAACTTTATG   1650

551  --------------------------------------------------    550

1651 GACCACACTCACCAATCCACACCCTTATTAATGCTCCGTTCGACATCATT   1700
```

551 --   550

1701 AGAATAACCAAGACATTCTATATAACTACGTGCTCGGAATATCCTAATAG   1750

551 --   550

1751 GAAAGTTGGAAGACTTTCCTACTGGAGTTGTGTAACTCCAGATGATGCCA   1800

551 --   550

1801 TAAAAAGCCCTTCCTCGGATTCGAACCGATGACTTTCTTAGCCTATTTCT   1850

551 --   550

1851 AAAGGGCGAACTGATCAACTTGCATGTGTTAAGCATATAGCTAGCGTTCC   1900

551 --   550

1901 TTCTGAGCCAGGATCAAACTCTTCTTTTGACCATGATTGAGCCCTGCAGT   1950

551 --   550

1951 GGTAGAGCCTAGTGAACCAGACATACGACTGGAAACCTGAATAGCCCTTC   2000

551 --   550

2001 TTCTCTTATGAGATTTCGAGGCTAGCTTTTTCTCGTACTATACCATCTAT   2050

551 --   550

2051 ACCATCCAAAGAAGTAAGGAGAATTCTCATCCCGGTGCCAAGTAAGCCAA   2100

551 --   550

2101 GCCGGCAGCGTCAGGGATAGGGTACGGGTCTTTCTAATGAATTTACATAC   2150

551 --   550

2151 TGATGAGCCAAGATCAAAAGCTAGAAAGGGTATTTTGGTAGTAAAGAGAG   2200

551 --   550

2201 AACCCCGATCAGTAGATAGAGATGGCCTTTAGCAAGAGGCTCAACCGATA   2250

551 --   550

2251 GTCCAAAGGTTCTAGACCCAGACGGTTATAACGAGACTAATAAGTAGAAA   2300

551 --   550

2301 GTACACGCCCCTTGCATTTTACTCAAGCCCTAGAAACGTCTGAAGCCTAT   2350

551 --   550

2351 GCTTTTGCTTCGGAGTTCGTGTCGGCGGGAGGGTCCTCGTTCATTTGTTT   2400

551 --   550

2401 CCAAAACTATAGGAGAAGGGTATCCCATTTTATGAAGCGGATTCGACTGG   2450

551 --   550

2451 AGATGGGTATTCAAATGTAGATTCAGTAGATTCAGTAGATTCATATGCGT   2500

```
551 --------------------------------------------------   550

2501 CCGCCTGTATCCGCAGATGAAGATGAGGATTTAGCTTCTTCAGGAGATTA   2550

551 --------------------------------------------------   550

2551 TGGAACAACGGATTCTTATTCTTTTGATGGCGGGTGAAACAGTGGGCAAT   2600

551 --------------------------------------------------   550

2601 TCTATATTATATAGTCTAATTGAGAAGGGCATGCGTCTTTTTCTGCGCCG   2650

551 --------------------------------------------------   550

2651 GTTTTGCTTATCTTTTGCAGATACGTCTACTTTCTCTCCTTTGGTTTGCG   2700

551 --------------------------------------------------   550

2701 GCCCCCAACTGGAGCTATTGCTTGTCGGCTTCGGGCTCTTAGCCAACGGT   2750

551 --------------------------------------------------   550

2751 TACCGTCAAACCCTTCCACGGATCACTCACCTACTTACCCTGAGACCAAT   2800

551 --------------------------------------------------   550

2801 GATCTTGAAGCAGAACCAGCTCCACTGATGATCATCGCGAAATCACACTA   2850

551 --------------------------------------------------   550

2851 AGAAAGGTAGATGGGAACAGCGCATAAACGGCTTATTCCTAATTCCTAAG   2900

551 --------------------------------------------------   550

2901 GTAAAAGGGTTTCCCTTAGATTATGCCTTTCCACTAGACTAAAAGAAGAC   2950

551 --------------------------------------------------   550

2951 GGTGACTCTACGAGGAATCAGGCTTATAAAAGAAAAGGAGGGACTCGAAA   3000

551 --------------------------------------------------   550

3001 TTGGTTATCAGTCAGGTCGTGGGAGACTAGGGAGTTTAAGTGGATTCCGT   3050

551 --------------------------------------------------   550

3051 AGCAAGAGCTAGCCGAATATATCCTATATCTATCTTTCTTGCGAAGGTCC   3100

551 --------------------------------------------------   550

3101 TCGACGCATCAGCTACATAATATCTGTAAAAATCTTGCCACTGGCGAACC   3150

551 --------------------------------------------------   550

3151 ATAATCTCTGGTGCTATCGACCTTTCGGAAAGGCTGACTTGAACACAAAG   3200

551 --------------------------------------------------   550

3201 CCAATCTTCGAGAGGTTAGTGGATAACTGTAACCAGTTCGTCCTTCTTCG   3250

551 --------------------------------------------------   550
```

3251 GAGAAATCGGGGAGCGGGCTTAAGGCTAGGGCCCCTCCGCGCATCAGAAA 3300

551 -- 550

3301 TCCTTGCTTACGATCTGGAGAGAAGGATCTTTTCCAAGAAAGACCTATTG 3350

551 -- 550

3351 CCCTTATTACTCCAAGAGATTCAAAGCGGCTTTAGATTAGTTGATTAGAC 3400

551 -- 550

3401 AATTCCTACTGCTGTGCTAAAGGCTAGAGAGGCAAGAAGAACATCTTACT 3450

551 -- 550

3451 CAACAAGAGTATCGGAAGAGATGCGCCTTCGGGACTTCCCTTTTAGCGTG 3500

551 -- 550

3501 GGAGTAGAGAAGGAAAAGGCGAAGCTGAGTCATATAATAAGTAAGGGAAC 3550

551 -- 550

3551 AAAGTGAGCTATAAGGCGATTGAATTCATTTCAGTTTAGTTGATATGAAA 3600

551 -- 550

3601 GCTAGCAGAGCTCATATCGCATTTGAAAGCAAGTGAGATATCACTATTGA 3650

551 -- 550

3651 TTGATTGATTGATAGAAAGCCCTATTGTAGACTAATAGCTCTAGCTTTCG 3700

551 -- 550

3701 ATTTGTTAGGAAGAGCTATTATTAATAGTAATTTCCCGCTTCAAGCAAAG 3750

551 -- 550

3751 TCAAGGACCTTTCCTCATTGGCCTTCGGCTTCAGCAAATTGATCTGGGAC 3800

551 -- 550

3801 CTTGTTTCGGTATAAGGACCAAGCCTTCGAGAGTCATCCTTCTTGCTACT 3850

551 -- 550

3851 TTGTTTTGTTAGAGGGGGTTCTTTAGACTCTCGGAACAAGGCTGTTGGTT 3900

551 -- 550

3901 GTTTCTCAGTCAGGCATGTCTGTGCTGCGCCAACGGGGATGGAGGAGATG 3950

551 -- 550

3951 CCCCGTCAGGAGGTGTTATGCGCAGACCACTCAATCTTTTGGAAACTGGG 4000

551 -- 550

4001 TCCGCTGGGGCGGGTTCTTTGGTTAGCACACTTATCTGGGAGCTTTGTTT 4050

551 -- 550

4051 TTCTTCATGACTGCCGATCATAATTCACTTCGCATCCATCAAGCTCTGAA 4100

551 -- 550

4101 CCTTCATTTTGAAATCTTCGCAATCTCGGATGGAATGACCTTTTGCTCCT 4150

551 -- 550

4151 TGATGAAACTCACATGATTCATCTTTTTCATAACTAGTTACTTGAGATTG 4200

551 -- 550

4201 CTCTTCGTAAAGTTCTGGTTGGACATAATGAGTGTAACGAAAAGAAAGAA 4250

551 -- 550

4251 AGACTAGAAGACATAGGGAATTTCAAGTAATACGTATTCGTTTATCAACT 4300

551 -- 550

4301 CATTTCGTCTCACTACGTATAGGGAATAGGGAAAAGCCTTGCCTTATTCA 4350

551 -- 550

4351 AGAGTACCAGGAAGGAGAAGAATAAGCAGAATTGCTTAAGTAAGGTAGTC 4400

551 -- 550

4401 GCTTCTTTTTTTCGGTATACCGCTCTGCGAGCAGAGCGAATTAACATAAA 4450

551 -- 550

4451 TCTTTCCGA*ATGAATATCTTTGATATTTTCACGATTGCCTATTCAATGAT* 4500

551 -- 550

4501 *TTTTTTTGTCTCTTTCACTTTTTTCGTAGCCAGACACAAACTTTCTTCCT* 4550

551 -- 550

4551 *ATTTATCCTTTAATGACCTTCTGACTAGAGAAGAATACCTTCGGCTGCTT* 4600

551 -- 550

4601 *GTCCCGGAGGCCCTTCAGAAGGATATTCAACTAATACTAAACTTATATTT* 4650

551 -- 550

4651 *GACTAACTTGGGCGAGAGGATGCCCCAAGGGCACTCTTGGCAAGAGCTCG* 4700

551 -- 550

4701 *CCCACAAAATTCACGAGGGCTCTCAAGACAAGGAGTTCCTTCTTTCCATT* 4750

551 -- 550

4751 *ATATCTGACATTGTTTCTAATGGAATGTCAAGTGAATGGGTCTTGATGGC* 4800

551 -- 550

4801 *TTTGCAAATCCTGAAAGATTGGACCTTTCCTCTATAA*TAGAAGGATATTT 4850

```
 551 --------------------------------------------------   550

4851 CAAGTATGGAAGAAATCTTATTTTTTAGCCTCATTACTTCCCACTGGGCG   4900
                                 ||||||||||||||||||||||
 551 ----------------------------CCTCATTACTTCCCACTGGGCG   572

4901 AGAGGTGCACCTAACCCACCTACCCACTCATCAATCACGGTGTTCAGCTG   4950
     ||||||||||||||||||||||||||||||||||||||||||||||||||
 573 AGAGGTGCACCTAACCCACCTACCCACTCATCAATCACGGTGTTCAGCTG   622

4951 ACTTGCACTGATAGACCCCTATTGTATTGGAATTAGGCGCCCATCTTTTT   5000
     ||||||||||||||||||||||||||||||||||||||||||||||||||
 623 ACTTGCACTGATAGACCCCTATTGTATTGGAATTAGGCGCCCATCTTTTT   672

5001 ACTGTTGTCAATGAATCTCTTCCATGTTCGATTCTACTCTATGTTAGAAC   5050
     ||||||||||||||||||||||||||||||||||||||||||||||||||
 673 ACTGTTGTCAATGAATCTCTTCCATGTTCGATTCTACTCTATGTTAGAAC   722

5051 ATTTCTGTGAATGCTATTCTGATCTAAGTGGTCTTATTCTTTGTCCCGTG   5100
     |||||||||||||||||||||||||||
 723 ATTTCTGTGAATGCTATTCTGATCTAA                          749

5101 C   5101-P3

 750      749
```

Additional file 3 Alignment of sequences of PCR products obtained after amplification with: **A.** P6 and P3, **B.** P7-P3 and **C.** P11-P3 primers (in bold, P3 complementary strand) in SH9B (upper sequence) and representative edited plants (**A.** T2-10, **B.** T1-49, **C.** T2-12, lower sequences). The green, blue, and brown fragments indicate *orf125*, the first part of *nad4*, and the last part of *orf247*, respectively. Orange and red sequences indicate the regions targeted by TALEN1 and TALEN2 pairs, respectively (site for *Fok1* cut underlined). The magenta and yellow sequences represent the DR19 and DR14 repeats putatively involved in the recombination determining a 236 bp and a 1066 bp deletion, respectively.
