# Supplementary material for: Mitochondrial DNA editing in potato through mitoTALEN and mitoTALECD: molecular characterization and stability of editing events
Source: Plant Methods. 2024 Jan 5;20:4. doi: 10.1186/s13007-023-01124-9 (PMC10768376; doi:10.1186/s13007-023-01124-9)
Supplement: Supplementary file 5 — Additional file 5. Sequence and position of direct repeats (DR) in the SH9B genomic region comprised between primers P11 (orf247) and P3 (nad4 exon 1). [file 13007_2023_1124_MOESM5_ESM.pdf]

**Additional file 5** Sequence and position of direct repeats (DR) in the SH9B genomic region comprised between primers P11 (*orf247*) and P3 (*nad4 exon 1*).

| Code | Sequence (5'→3') | Position[a] (nucleotide) | Length[b] (bp) |
|---|---|---|---|
| DR1[c] | TCAGAAATCC | 359, 3294 | 10 |
| DR2 | AGAATACCTT | 417, 4582 | 10 |
| DR3 | GGGAGACTAGGGAGT | 581, 3021 | 15 |
| DR4 | TAATATACCA | 595, 615 | 10 |
| DR5 | TATACCATCT | 598, 2039 | 10 |
| DR6 | CTTTTAAATA | 761, 1193 | 10 |
| DR7 | CTTTTCCAAG | 805, 3329 | 10 |
| DR8 | TTGCCTTATTCA | 965, 4339 | 12 |
| DR9 | ACTTTCTTAG | 976, 1832 | 10 |
| DR10 | CTATACCATC | 2038, 2047 | 10 |
| DR11 | TCTTATTCTTT | 2564, 5082 | 11 |
| DR12 | ATTGATTGATTGAT | 3646, 3650 | 14 |
| DR13 | CCCTATTGTA | 3670, 4967 | 10 |
| DR14 | GGACCTTTCCTC | 3755, 4821 | 12 |
| DR15 | TTTCGGTATA | 3805, 4410 | 10 |
| DR16 | ATTTCAAGTA | 4270, 4847 | 10 |
| DR17 | GAGAAGAATA | 4365, 4578 | 10 |
| DR18 | AGAAGGATATT | 4617, 4839 | 11 |
| DR19 | TGGGCGAGAGG | 4659, 4895 | 11 |

[a] Referred to the amplicon P3-P11
[b] bp, base pairs
[c] Repeat order and sequence refer to the coding strand
