# Supplementary material for: Mitochondrial DNA editing in potato through mitoTALEN and mitoTALECD: molecular characterization and stability of editing events
Source: Plant Methods. 2024 Jan 5;20:4. doi: 10.1186/s13007-023-01124-9 (PMC10768376; doi:10.1186/s13007-023-01124-9)
Supplement: Supplementary file 7 — Additional file 7. Codon changes in regenerated plants after base editing of orf125 with TALECD1 and TALECD2 pairs. [file 13007_2023_1124_MOESM7_ESM.pdf]

Additional file 7 Codon changes in regenerated plants after base editing of *orf125* with TALECD1 (**A.** D1) and TALECD2 (**B.** D2) pairs. Modified C/Gs in the coding strand are numbered from the 5' start of the spacing window.

A. *Site 1 (D1)*

	**Locus (SH9B)**	
**Plant**	**5'-(AAG)G_5(ATA)-3'**[a,b]	**5'-(ATT)C_{11}(AAC)-3'**
D1-5	GAT→AAT	CAA→C/TAA[c]
D1-20	-[d]	-
D1-28	GAT→AAT	CAA→C/TAA
D1-36	-	-
D1-48	GAT→AAT	CAA→C/TAA
D1-51	GAT→AAT	-
D1-54	GAT→AAT	CAA→C/TAA
D1-57	GAT→AAT	CAA→C/TAA
D1-61	GAT→AAT	CAA→C/TAA
D1-68	GAT→AAT	CAA→C/TAA
D1-69	GAT→AAT	CAA→C/TAA
D1-70	GAT→AAT	-
D1-71	-	-
D1-84	GAT→AAT	CAA→TAA
D1-85	GAT→AAT	-
D1-90	GAT→AAT	CAA→C/TAA
D1-93	GAT→AAT	CAA→C/TAA
D1-94	-	-
D1-98	-	-
D1-103	GAT→AAT	CAA→C/TAA
D1-113	GAT→G/AAT	-

B. *Site 2 (D2)*

	**Locus (SH9B)**				
**Plant**	**5'-(CAC)G_2(AGG)-3'**[b]	**5'-(CGA)G_4(GGC)-3'**[b]	**5'-(GAG)G_5(GCT)-3'**[b]	**5'-(GCT)C_9(TCA)-3'**	**5'-(TCT)C_{11}(AAG)-3'**
D2-1	-	-	-	-	-
D2-3	-	-	-	-	-
D2-9	-	-	-	-	-
D2-13	-	-	-	-	-
D2-16	-	-	-	-	-
D2-17	-	-	-	-	-
D2-18	-	-	-	-	-
D2-19	GAG→G/AAG	GAG→GAG/A	GGC→G/AGC	TCT→TC/TT	CAA→TAA
D2-20	GAG→G/AAG	-	-	TCT→TC/TT	CAA→TAA
D2-21	-	-	-	-	-
D2-22	GAG→G/AAG	GAG→GAG/A	GGC→G/AGC	TCT→TC/TT	CAA→TAA
D2-23	-	-	-	-	CAA→TAA
D2-24	GAG→G/AAG	GAG→GAG/A	GGC→G/AGC	TCT→TC/TT	CAA→TAA
D2-25	GAG→AAG	GAG→AAG/A	GGC→G/AGC	TCT→TC/TT	CAA→TAA
D2-27	-	-	-	-	CAA→C/TAA
D2-29	-	-	-	TCT→TC/TT	CAA→TAA
D2-32	-	-	-	-	CAA→C/TAA
D2-33	-	-	-	-	-
D2-34	-	-	-	-	CAA→TAA
D2-37	-	-	-	-	-
D2-38	-	-	-	-	-
D2-39	-	-	-	-	-

[a] The number (subscript) indicates the position of Cs or Gs in the 15 bp window between the two TALEs
[b] Complementary strand
[c] Heteroplasmic base change
[d] No base change
